# Damage and reliability analysis of double-arch tunnel without a middle pilot tunnel under blast load

**DOI:** 10.1038/s41598-024-59681-5

**Published:** 2024-04-22

**Authors:** Bingxi Jian, Tiejun Tao, Shuai Song, Caijin Xie, Xingchao Tian, Guoqing Li, Antong Wan

**Affiliations:** 1 College of Civil Engineering, Guizhou University, Guiyang, 550025 China; 2 College of Mining, Guizhou University, Guiyang, 550025 China; 3 College of Architectural Engineering, Guizhou Polytechnic of Construction, Guiyang, 551499 China

**Keywords:** Tunnel blasting, Finite element, Double-arch tunnel, Surrounding rock, Damage, Civil engineering, Petrology

## Abstract

In this study, a new type of multi-arch tunnel construction method is proposed. This effort is undertaken due to the many disadvantages of the traditional multi-arch tunnel construction method. Furthermore, this method omits the construction of a middle pilot tunnel, and it has the advantages of safety, high efficiency, and being economical. When using the method of continuous arch tunneling without a middle pilot tunnel, the blasting of the first tunnel and the following tunnel has a greater impact on the surrounding rock damage, as well as on the supporting structure of the same cross-section. Therefore, this study uses LS-DYNA finite element software to construct a three-dimensional numerical model. In addition, the perimeter rock damage law and mechanical response characteristics of the supporting structure in the same cross-section of the first tunnel, as well as the following tunnel after blasting without a middle pilot tunnel, are analyzed. At the same time, the results of the study are based on optimizing the blasting program, and these are then applied to the field. Through the results, it is found that, after blasting a continuous arch tunnel without a medial pilot tunnel, the surrounding rock damage in the arch cross-region of the double-arch tunnel (where the arch top and the arch shoulder are more significant) and the effective stress of the supporting structure exceed the strength design value. In addition, the maximum adequate pressure is distributed in the medial diaphragm wall. With the optimized blasting scheme, the range of the peripheral rock damage is reduced by a maximum of 67%, and the effective stress in the supporting structure is reduced by 25.9–64.8%. The research results are of great significance in terms of improving construction safety, economic efficiency, and project quality, as well as in promoting the research and development of new work methods for double-arch tunnels.

## Introduction

The number of tunnel constructions has gradually increased with the rapid development of transportation infrastructure. The construction of a geographic environment is also becoming increasingly complex due to construction within terrain and space constraints, which has resulted in producing a new form of tunnel structure. Double-connected arch tunnels have also been born as a result. Compared with ordinary tunnels, its advantages are obvious, but the construction process is complicated. The left and right tunnels are close to each other, and respective excavation efforts interfere with one another^1,2^. When a blasting excavation is used, the surrounding rock’s damaged state and the supporting structure’s stress state become more complicated, thus increasing the difficulty in the construction and design^3,4^.

Due to the unique characteristics of the structure of a continuous arch tunnel without a middle pilot tunnel, its use in blasting excavations to produce damage to the surrounding rock law and to ordinary detached tunnels represents a big difference^5^. Ordinarily separated tunnels have their left and right tunnels farther away, and the cumulative effect on the surrounding rock is small. When the distance between the left and right tunnels of separated tunnels reduces, the cumulative damage produced by the surrounding rock between the left and right tunnels also increases. The peripheral rock damage law of blast excavations of a continuous arch tunnel is extremely complex and complicated, and engineers need to fully understand the peripheral rock damage law and the dynamic response law of the supporting structure of the continuous arch tunnel under the action of a blasting load. It should be noted that a too extensive range of damage to the surrounding rock will lead to poor stability in the tunnel’s surrounding rock. Moreover, excessive over- and under-excavation increases the construction consumables and construction risk; after the completion of construction, this may exacerbate the situation of eccentric force in the continuous arch tunnel, which will lead to cracking and the destruction of the supporting structure^6–9^. Therefore, it is of great significance to study the damage law of blasting on surrounding rock in the construction of tunnels without a middle pilot tunnel, as well as the dynamic response of the supporting structure for efficient, safe, and economic construction.

To ensure that the project can be completed safely and efficiently, it remains an urgent problem to determine the dynamic characteristics of the surrounding rock during a blasting and excavation process^10^. Certain scholars have carried out research from two aspects on this topic. One aspect is numerical simulation calculation. Xue et al.^11^ used FLAC3D finite element numerical simulation software to research the construction method of continuous arch tunnels to explore the rule of change in surrounding rock deformation and stress. Wu et al.^12^ used ANSYS/LS-DYNA finite element numerical simulation software to study the damage effects of small proximity tunnel blasting and excavations on interbedded rock and supporting structures. Lei et al.^13^ used ANSYS/LS-DYNA finite element numerical simulation software to simulate the tunnel blasting contour of weak interbedded layers, as well as put forward an optimized blasting solution for the damaged condition of surrounding rock. Du et al.^14^ used numerical simulation software to analyze the dynamic response of an existing tunnel structure in comparison with the blasting and excavation of new tunnels. Ling et al.^4^ used ANSYS/LS-DYNA finite element numerical simulation software to simulate the cumulative process of millisecond-delayed blasting damage to the surrounding rock, and they also analyzed the cumulative damage evolution law of the rock mass. Gao et al.^15^ used FLAC3D finite element numerical simulation software to simulate different methods of excavation in a continuous arch tunnel, analyzed the stability of a shared middle wall, and also determined an optimal scheme of excavation. Kang et al.^16^ used ANSYS AUTODYN finite element numerical simulation software to simulate the scope of influence with respect to tunnel blasting damage, and they put forward a design of blasting that can be applied to actual tunnels. Yoo and Choi et al.^17^ carried out a study on the effect of construction sequences on triple-arch tunnels using numerical studies to investigate the surface displacements and shotcrete stress changes. There are also many other scholars who have conducted numerical simulation software optimization via the optimization of software algorithms, which were used to make the calculation results more accurate^18–21^. Some of these scholars achieved this through the establishment of unique numerical models to simulate certain special interaction relations^22,23^, as well as to help make the results of the simulation calculations be closer to reality. Analyzing numerical simulation results is also vital; certain scholars, through introducing the Laplace–Fourier transform and other analytical theories, have relied on processing results to obtain more accurate results^24–27^. The other aspect is to conduct research from field experiments. Huang et al.^28^ used similar scale model experiments to simulate tunnel blasting, as well as explored the attenuation mechanism of explosions in tunnels when under near-field blasting conditions. Cao et al.^2^ used on-site ultrasonic experiments to analyze the process of vibrational damage accumulation in rocks that were shared by neighboring tunnels, as well as investigated the accumulation of damage in the rocks that were subjected to multiple blasting loads. Li et al.^29^ used the on-site monitoring of vibration signals of multiple blasts to analyze the damage evolution of the surrounding rock for the purpose of studying the cumulative damage law of the surrounding rocks of tunnels that had been subjected to multiple blasts. Zhong et al.^30^ used the on-site monitoring of multiple blasting vibration signals to analyze the damage evolution of surrounding rock in order to study the cumulative damage law of the surrounding rock of a tunnel that had been subjected to multiple blasting. Liu et al.^31^ studied the structural dynamics of small-clearance tunnel blasting excavations, monitored blasting vibration velocity, and analyzed the blasting vibration attenuation law through on-site blasting. Qiu et al.^32^ carried out physical modeling experiments to study the influence of different blasting locations on the surrounding rock of tunnels.

In recent years, despite the majority of scholars having investigated the mechanical behavior of multi-arch tunnel construction under various excavation methods to assess tunnel structural stability and refine construction techniques, the focus of most studies has been on surrounding rock failure, stress in small-clearance tunnel drilling structures, and traditional blasting excavation methods. There is a lack of research on the construction techniques for multi-arch tunnels, particularly regarding the effects of blasting excavation without a middle pilot tunnel on surrounding rock damage and the mechanical properties of support structures. The construction method for double-arch tunnels without intermediate pilot tunnels presented in this study, especially the groundbreaking pre-embedding of connection points for steel arch frames in the primary support structure of the first tunnel, is a novel contribution by our team, as detailed in referenced publications^1^. We have explored the failure patterns of surrounding rock under this method from a new perspective, as well as the dynamic characteristics of support structures in the same section of left and right tunnels after continuous blasting without a middle pilot tunnel. Based on the engineering example of the Xiqu Tunnel in Yunnan Province and using the LS-DYNA finite element software, a three-dimensional numerical model for simultaneous sectional blasting excavation of the leading and trailing tunnels was constructed. This model was used to analyze the failure patterns of surrounding rock in no-middle-pilot-tunnel double-arch tunnels and the mechanical properties of support structures. According to the research findings, we have not only optimized the blasting scheme and successfully applied it to practical engineering verification but also provided valuable references for the design and construction of similar projects.

## Definitional theory of perimeter rock damage

The damage variables need to be defined before the damage analysis of the surrounding rock can be undertaken. How the destruction of rock occurs and the specific manner in which it is damaged is closely related to the microstructure and macro mechanics of rock; as such, the macro mechanics should be fully considered when defining the damage variables of rock. Therefore, when defining the damage variables of rocks, we should start by considering the following two aspects: (1) The damage of rocks is determined by the expansion and development of cracks, which are often only observable from a microscopic point of view. As such, we should consider factors such as the number, length, and volume of cracks^33–35^. (2) From the macroscopic point of view, when the damage of rocks occurs, its mechanical parameters will also change. As such, we should consider factors such as the elastic modulus and permeability of the rock. When the rock undergoes damage, the development of microscopic cracks leads to the deterioration of its macroscopic mechanical properties. Among them, the change in the rock's acoustic wave velocity can effectively demonstrate its degree of damage.

The damage expression defined in terms of the change in the elastic modulus of the rock^36^ is as follows:

1 $$ D = 1 - E/E_{0} $$

where *E*_*0*_ is the initial modulus of elasticity (MPa) before blast damage, and *E* is the modulus of elasticity (MPa) of the rock mass after blast action.

According to the elastic wave theory, the elastic modulus of the rock mass before and after blasting action can be expressed by Eqs. (2) and (3):

2 $$ E_{0} = \rho_{0} \nu_{0}^{2} (1 - \mu_{0} )(1 - 2\mu_{0} )/(1 + \mu_{0} ) $$

3 $$ E = \rho \nu_{{}}^{2} (1 - \mu )(1 - 2\mu )/(1 + \mu ) $$

where *ρ*_*0*_ and *ρ* are the density of the rock body before and after the blasting effect, respectively; *μ*_*0*_ and *μ* are the Poisson's ratio of the rock body before and after the blasting effect; and *ν*_*0*_, *ν* is the wave velocity at the same location of the rock body when under a blasting effect.

When smooth surface blasting or pre-cracking blasting is used in tunnel blasting and excavation, it can be assumed that there will be no qualitative change in the reserved surrounding rock before and after blasting. Therefore, it can be assumed that the Poisson's ratio and density of the reserved surrounding rock of the tunnel are approximately equal before and after the blasting excavation. As such, it can be assumed that the Poisson's ratio and density of the reserved surrounding rock of the tunnel are approximately equal before and after blasting and excavation, that is, *ρ*_0_≈*ρ* and *μ*_0_≈*μ*. Combining the above Eqs. (1), (2), and (3) provides the following derivation:

4 $$ D = 1 - E/E_{0} = 1 - (\nu /\nu_{0} )^{2} $$

With the above content, the damage produced by the rock body under the action of blasting can be approximated by the change in the longitudinal wave speed before and after the rock body blasting. In practical engineering, we often use the acoustic wave velocity of the rock to determine the degree of damage to the rock. Therefore, the rate of change in wave velocity *η* before and after blasting the rock can be used to judge the degree of damage to the rock.

5 $$ \eta = \frac{{\nu_{0} - \nu }}{{\nu_{0} }} = 1 - \frac{\nu }{{\nu_{0} }} $$

Judgment criteria^4^: when the variation rate of the wave velocity is *η* ≤ 10%, the effect of blasting on the rock mass can be largely ignored; if it is 10% < *η* ≤ 15%, then the blasting effect on the rock produces a slight effect; and if it is 15% < *η*, then the rock has undergone a significant impact. When *η* > 10%, then the rock body (of which the damage threshold is *D*_*1*_ = 0.19) has been subjected to blasting damage.

## Numerical simulation analysis of tunnel blasting in western Yunnan Province

### Project profile

**Figure 1:**
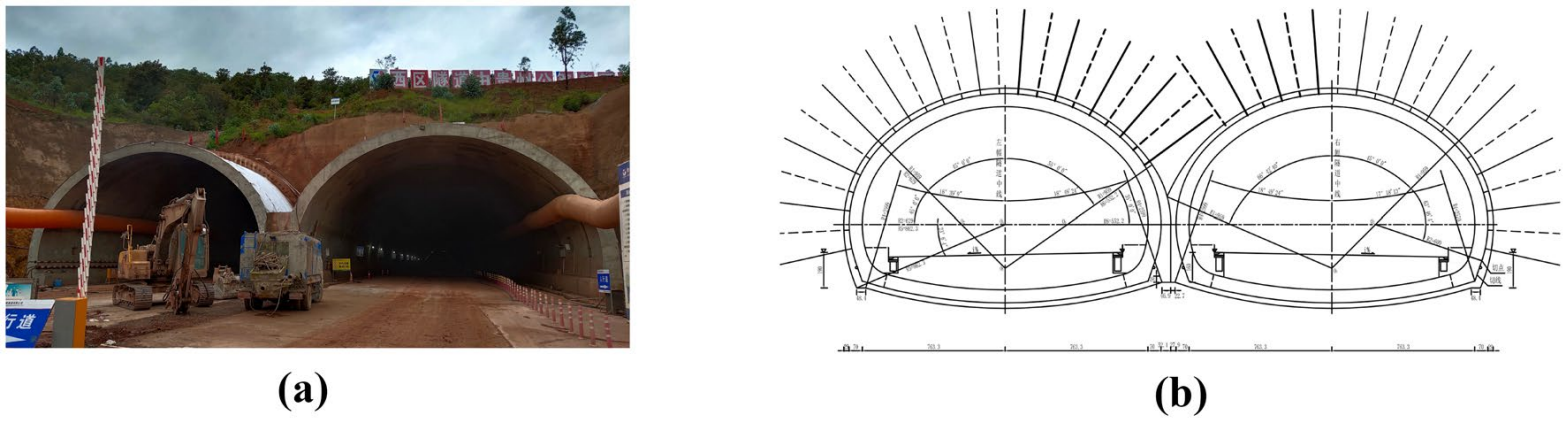
The cross-section of a double-arch tunnel. (**a**) Actual engineering drawings. (**b**) Design drawings.

The west tunnel is located in Dali Prefecture, Yunnan Province, and it has a large-span, two-way, six-lane, and double-arch tunnel. Its tunnel area elevation is 1765–1945 m, with a relative height difference of 74 m. As shown in Fig. 1a, the undulating topography of most of the tunnel slopes ranges from 15º to 25º, and the tunnel site area is characterized by steeper topography and a better-developed ground vegetation surface. The maximum depth of the tunnel is 60.76 m, the design speed is 80 km/h and the design gradient is − 1.7%. The surrounding rocks are mainly of class V, mainly sandstone, mudstone, dolomite and limestone, and the rocks are moderately weathered. Therefore, the geological report indicates that the surrounding rock is classified as Grade V in this study. However, the geological condition and integrity of the surrounding rock in our experimental section are better, and C25 concrete is used for initial support. The tunnel design is shown in Fig. 1b.

**Figure 2:**
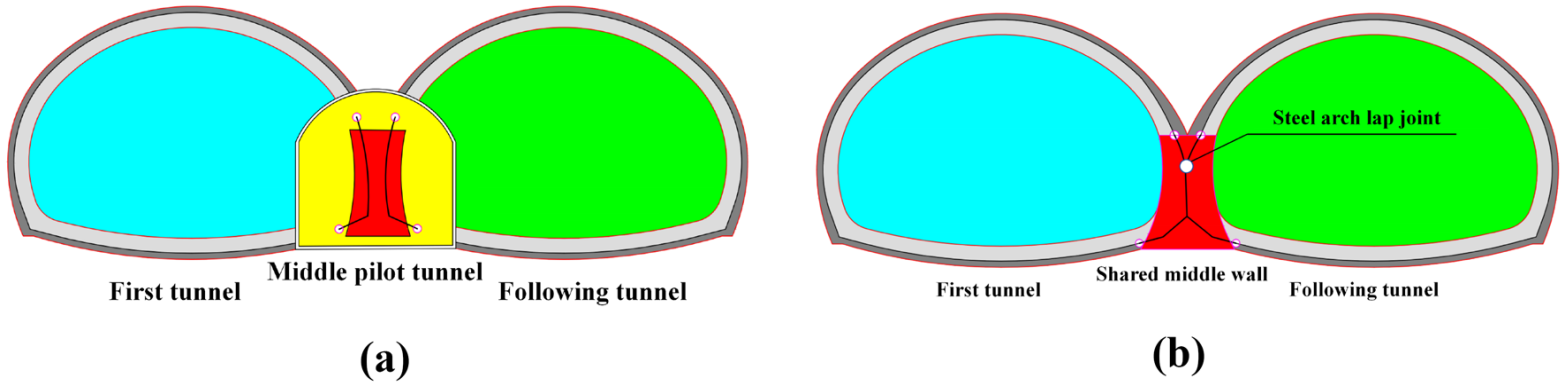
Two construction methods for double-connected arch tunnels. (**a**) Conventional double-arch tunnel. (**b**) Double-arch tunnel without a middle pilot tunnel.

This project adopted the double-arch tunnel construction method without a middle pilot tunnel. In highway tunnels, the tunnels are divided into two types: separated tunnels and continuous arch tunnels. The use of a continuous arch tunnel without a center guide tunnel is a new continuous double-arch tunnel construction method. As shown in Fig. 2a, in the traditional method of continuous arch tunnel construction, the middle pilot tunnel is constructed first, and the center wall is poured while the middle pilot tunnel is excavated. After the middle pilot tunnel is passed through, the first tunnel is next to be constructed and excavated; then, the following tunnels will be excavated after a certain excavation distance from the first hole has been achieved. Therefore, the traditional tunnel construction method of continuous arch tunnels is not only complicated and requires a long construction period, but it also generates a large amount of earth and rocks in the excavation process, which will also significantly impact the environment. There is a great deal of cultivated land at the inlet of the Xiqu tunnel in the district, and the use of separate tunnels not only fails to meet the requirements of highway alignment but also hurts cultivated land and forests, which is not in line with the concept of sustainable development. In contrast, the method of double-arch tunnel construction without a middle pilot tunnel results in the omission of an excavation of the middle pilot tunnel. Its excavation sequence is similar to that of the separated tunnel, as shown in Fig. 2b. The first tunnel is excavated, and the shared center wall is also poured at the same time when the first tunnel has its initial support carried out. The steel arch contact point of the initial support structure of the following tunnel is reserved on the other side of the center partition wall. The following tunnel is excavated at a certain distance from the first tunnel, and the steel arch of the following tunnel is directly connected to the reserved contact point of the first tunnel. Therefore, since there is no middle pilot tunnel, it dramatically reduces the tunnel footprint, which is suitable for environmental protection and savings in land costs. At the same time, the left and right tunnels share the steel arch frame for the initial support of the adjacent sidewalls, which reduces the construction steps and the amount of steel, as well as helps to significantly reduce construction costs.

Both construction methodologies exhibit distinct advantages and disadvantages. As illustrated in Table 1, in contrast to conventional construction approaches, the method employing continuous arch tunneling without the use of intermediate adits demonstrates superiority in the simplicity of construction steps and the reduction of construction timelines. However, the omission of intermediate adits significantly increases the impact of later stages of tunnel excavation on previously completed sections of the tunnel. Consequently, this approach requires more sophisticated blasting strategies and mandates the implementation of vibration damping mechanisms to mitigate the effects of such operations.

**Table 1:** Comparison of two construction methods.

Construction method	Advantages	Disadvantages
Traditional method	(1) The excavation of subsequent tunnels has minimal impact on preceding tunnels (2) Construction techniques are well-established (3) Smaller excavation cross-section, thus less disturbance to the surrounding rock mass	(1) The construction process is more complex (2) Longer construction period (3) Higher construction costs
Continuous arch tunnel without intermediate adit	(1) The construction process is simpler (2) Shorter construction period	(1) The absence of an intermediate adit reduces the distance between preceding and subsequent tunnel excavations, increasing mutual interference (2) The steel arch frames for subsequent tunnels are overlapped on reserved interfaces of preceding tunnels, which imposes higher requirements on the steel arch frames

### Original blasting program

**Figure 3:**
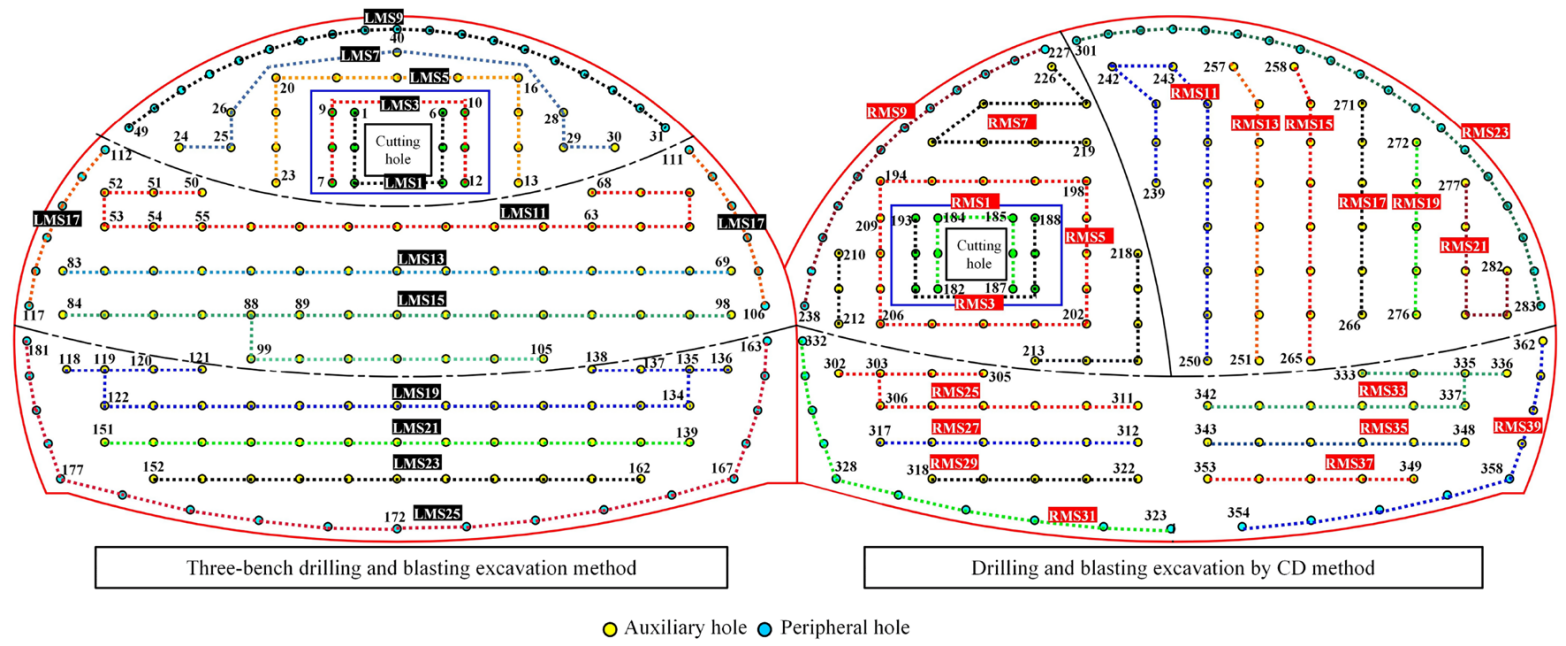
Schematic diagram of the blasthole layout.

For the first tunnel, we adopted a three-bench blasting excavation construction method; for the following tunnel, we adopted the CD method blasting excavation method. The layout of the holes and the detonation sequence is shown in Fig. 3. The cyclic footage was 1.5 m, the number of holes in the first tunnel was 181, and the number of detonation segments was 13; moreover, the number of holes in the following tunnel was 181, and the number of detonation segments was 20. The number of holes in the two blasting palm faces was 362 holes (which was achieved using millisecond-delayed blasting). The holes were 42 mm in diameter, and there were examples of uncoupled charging with a coupling coefficient of 1.31. The angle of the cutting hole in the first and following tunnel was the same, the angle of the cutting hole in the LMS1 and RMS1 segments was 57°, the spacing of the cutting holes was 50 cm, and the row spacing was 80 cm; furthermore, the angle of the cutting hole in the LMS2 and RMS2 segments was 59°, the spacing of the holes was 50 cm, and the row spacing was 80 cm. Lastly, the spacing of the peripheral holes was 72 cm. The blasthole parameters are shown in Table 2.

**Table 2:** Blasthole parameters.

Tunnel	Blasthole name	Blasthole diameter (m)	Detonator segment (MS)	Hole depth (m)	Blasthole number	Single hole charge (kg)
First tunnel	Cutting hole	0.042	1	1.5	1–6	1.50
	Cutting hole	0.042	3	1.5	7–8	1.50
	Upper step auxiliary hole	0.042	5, 7	1.5	13–30	1.350
	Upper step peripheral hole	0.042	9	1.5	31–49	1.350
	Middle step auxiliary hole	0.042	11, 13, 15	1.5	50–105	1.350
	Middle step peripheral hole	0.042	17	1.5	106–117	1.350
	Lower step auxiliary hole	0.042	19, 21, 23	1.5	119–162	1.350
	Lower step peripheral hole	0.042	25	1.5	163–181	1.350
Following tunnel	Cutting hole	0.042	1	1.5	182–187	1.50
	Cutting hole	0.042	3	1.5	188–193	1.50
	CDI auxiliary hole	0.042	5, 7	1.5	194–226	1.350
	CDI peripheral hole	0.042	9	1.5	227–238	1.350
	CDII auxiliary hole	0.042	11, 13, 15, 17, 19, 21	1.5	239–282	1.350
	CDII peripheral pore	0.042	23	1.5	283–301	1.350
	CDIII auxiliary hole	0.042	25, 27, 29, 33, 35, 37	1.5	302–322, 333–353	1.350
	CDIII peripheral pore	0.042	31,39	1.5	323–332, 354–362	1.350

### Numerical simulation methods

#### Three-dimensional numerical modeling

Among the existing numerical simulation methods, ANSYS/LS-DYNA has a significant advantage in calculating the large deformation dynamic response of nonlinear materials under the action of a high strain rate; thus, it is widely used in blasting simulations. In the numerical simulation of tunnel blasting, there are usually two methods through which to simulate the load generated by explosives: one is to use the equivalent load method, i.e., the load caused by the explosion of explosives when it is applied to an action surface; the other is to use the fluid–solid coupling method in LS-DYNA, which models the interaction between explosives, the air, and rocks, whereby the Lagrangian part is the rock and the explosives and air are represented by the Lagrangian–Eulerian (ALE) formula. Compared with the equivalent load method, it can simulate the evolution of damage to the rock after the explosion of explosives in each blasthole, and the simulation effect is closer to the actual site, which also facilitates the fine control of the blasting program. Therefore, this study adopts the fluid–solid coupling method, which can be used to fine tune the control of the blasting program.

**Figure 4:**
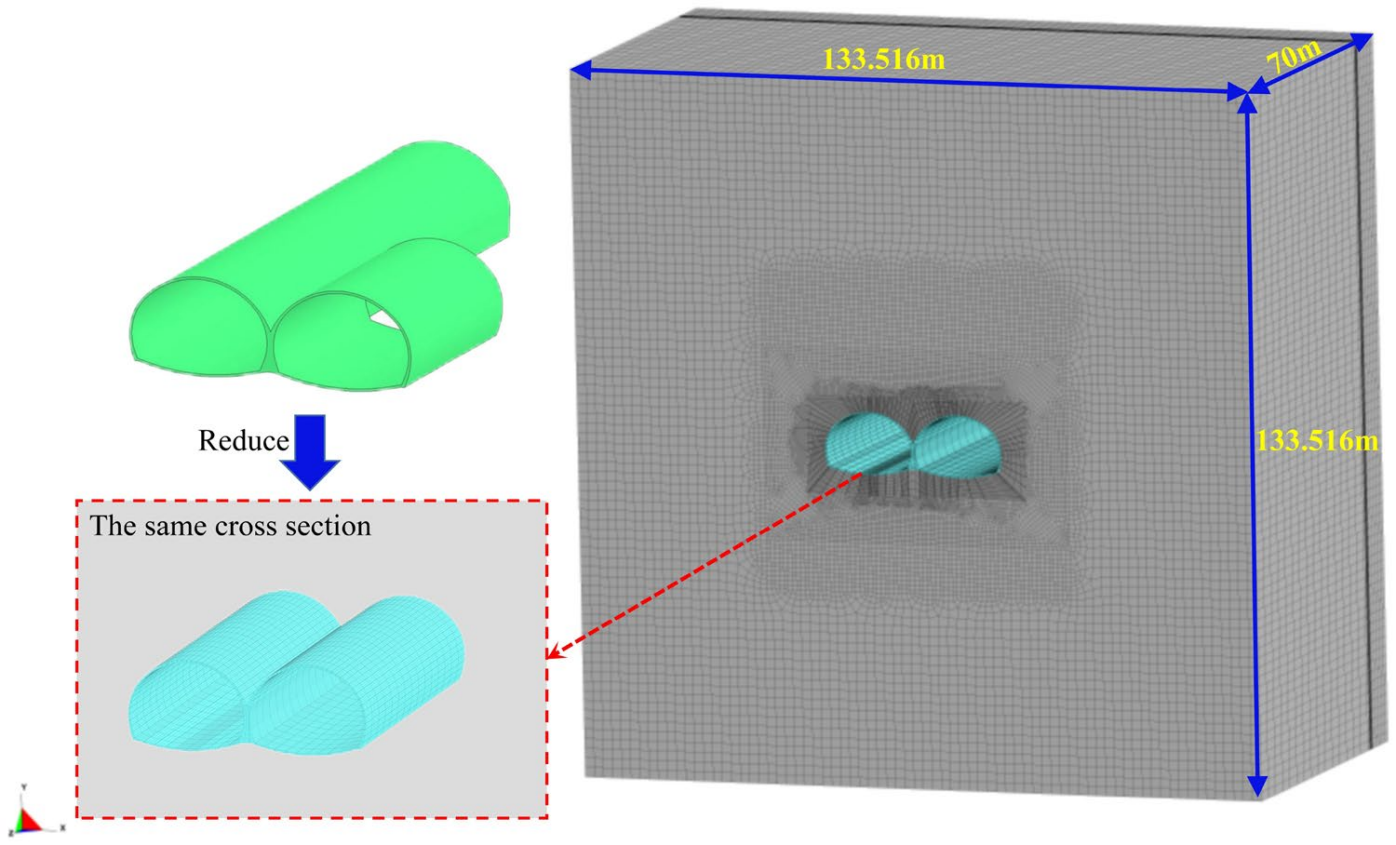
The three-dimensional numerical model.

To study the dynamic response law of the tunnel support structure and surrounding rock of the first and following tunnel of a continuous arch tunnel without a middle pilot tunnel in the same tunnel cross-section, taking into account the research objectives and simplification of this study, a three-dimensional numerical model is established as follows: the three-dimensional modeling and mesh delineation were carried out using AutoCAD and HyperMesh; thus, the three-dimensional numerical model was established, as shown in Fig. 4, according to the impact of the tunnel construction. According to the influence of the tunnel construction, the model size is usually considered to be 3–5 times the tunnel diameter; thus, the model size in this study was set at 70,000 × 133,516 × 133,516 mm, and there were 221,516 grid cells utilized. In addition, the palm surface of the first tunnel and the palm surface of the following tunnel were in the same cross-section. The model contains five parts: the initial support, explosives, the gun clay, air, and the surrounding rock.

#### Statics experiment and rock parameters

Based on the geological conditions, we have selected sandstone as the subject of study in this paper. The parameters were obtained through static experiments, and then validated through a combination of Split Hopkinson Pressure Bar (SHPB) tests and numerical simulations.

1. Experimental equipment and scheme
  The experiment employed the TAJW-2000 rock compression testing apparatus, as illustrated in Fig. 5. The system comprises a mainframe, hydraulic system, control unit, strain gauge, data acquisition unit, and a computer. The sandstone samples were subjected to uniaxial compression, triaxial compression, and Brazilian split tests. The experiments utilized a constant displacement rate of 0.005 mm/s as the loading control method. The triaxial experiments primarily adopted confining pressures of 5 MPa, 10 MPa, and 15 MPa.

**Figure 5:**
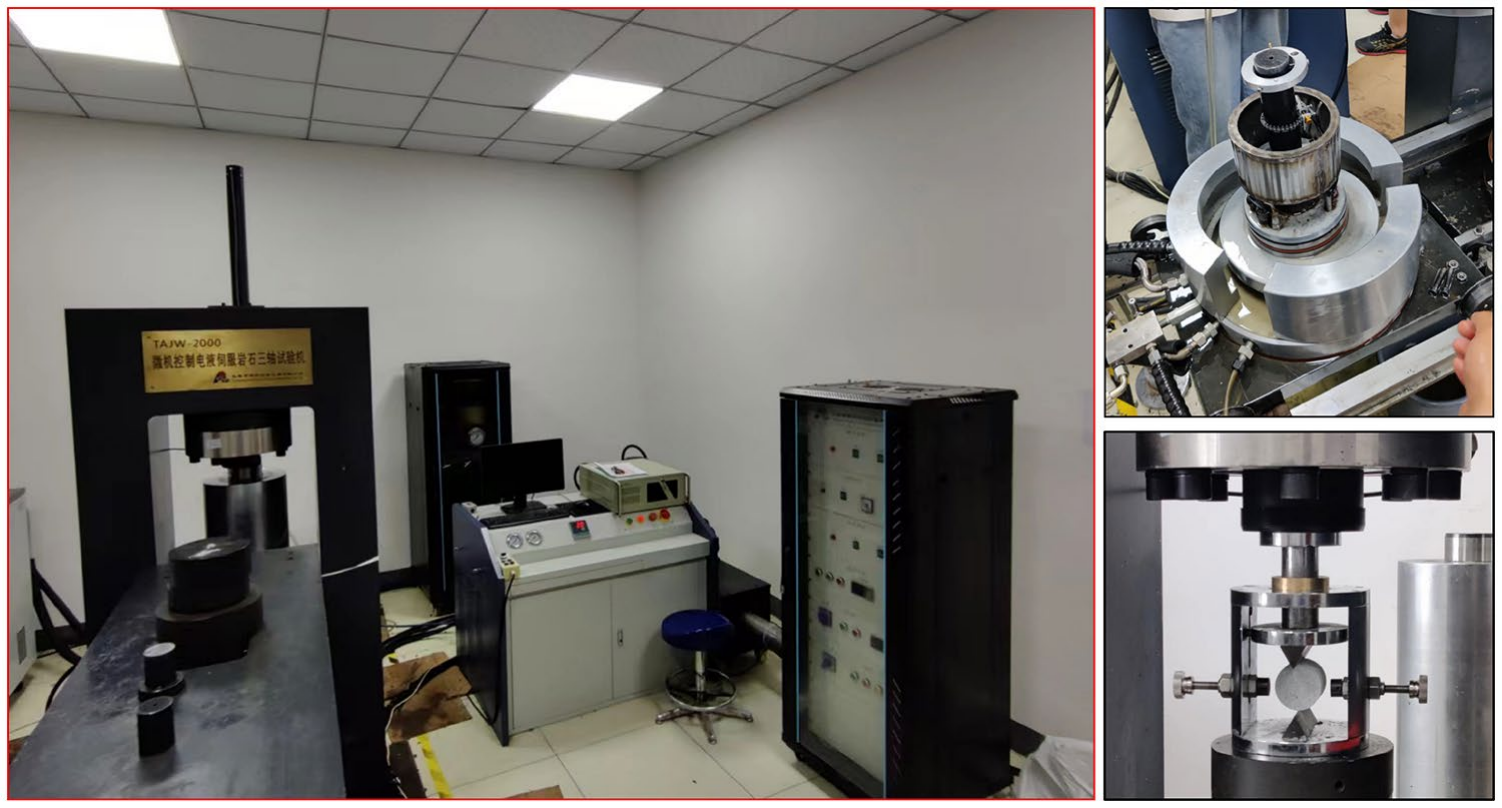
TAJW-2000 rock compression testing apparatus.
2. Static rock samples
  Samples were collected from the field and processed in the laboratory according to the experimental requirements of the International Society for Rock Mechanics to create standard-sized specimens, as shown in Fig. 6. The dimensions and weight of these specimens were measured, with the specific values presented in Table 3.

**Figure 6:**
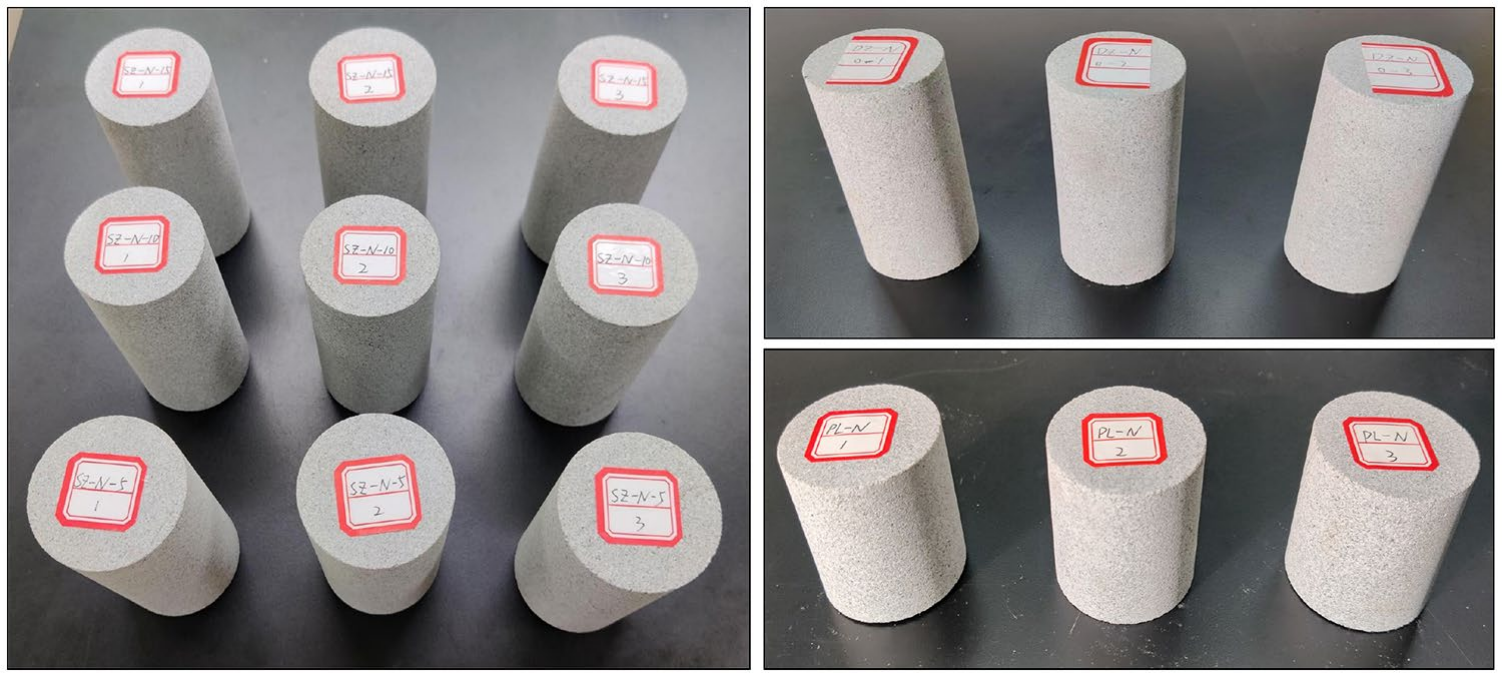
Rock specimens.

**Table 3:** Sandstone dimensions statistics.

Serial number	Diameter (mm)	Height (mm)	Weight (kg)	Volume (mm^3^)	Density (kg/m^3^)
DZ-N-0-1	49.90	100.26	490.20	195,973.996	2501.352271
DZ-N-0-2	50.14	100.12	491.70	197,587.3592	2488.519518
DZ-N-0-3	49.77	100.33	492.20	195,090.3332	2522.933822
SZ-N-5-1	50.26	100.07	491.60	198,435.114	2477.384118
SZ-N-5-2	50.20	100.16	491.30	198,139.657	2479.564199
SZ-N-5-3	50.20	100.09	490.50	198,001.1808	2477.257953
SZ-N-10-1	50.11	100.07	492.50	197,252.43	2496.800674
SZ-N-10-2	50.09	100.05	491.10	197,055.6144	2492.189839
SZ-N-10-3	50.01	99.84	492.30	196,014.3822	2511.550399
SZ-N-15-1	50.46	100.02	490.60	199,917.5861	2454.011223
SZ-N-15-2	50.08	100.13	491.80	197,134.4445	2494.74414
SZ-N-15-3	50.07	100.31	490.90	197,409.9643	2486.703251
PL-N-1	49.76	50.18	245.10	97,535.12774	2512.94078
PL-N-2	50.18	50.32	246.30	99,465.30144	2476.240422
PL-N-3	50.04	50.37	247.7	99,009.35006	2501.783921
Average value	–	–	–	–	2491.598435
3. Experimental results
  The static experiments conducted on the rock samples yielded the basic mechanical parameters as illustrated in Table 4.

**Table 4:** Basic uniaxial mechanical parameters of rock.

Density (kg/m^3^)	Compressive strength (MPa)	Elastic modulus (GPa)	Cohesion (MPa)	Friction angle (°)	Poisson's ratio
2491	71.67	4.723	18.72	34.84	0.24

#### Validation of rock material parameters

To validate the suitability of the RHT model parameters for sandstone, standard specimens of sandstone were prepared from cores drilled on-site and processed as shown in the figure. These specimens were then subjected to Split Hopkinson Pressure Bar (SHPB) experiments. Additionally, a 3D numerical impact model of SHPB was established using ANSYS/LS-DYNA finite element software. The applicability of the rock parameters was verified by comparing the experimental results with the numerical simulation outcomes.

**Figure 7:**
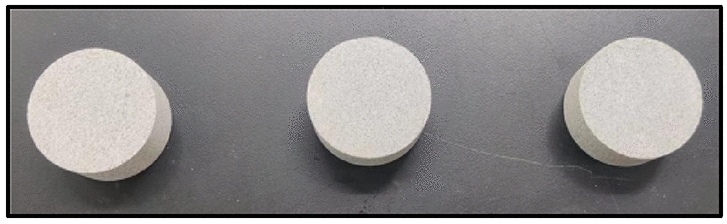
Sandstone sample.

**Figure 8:**
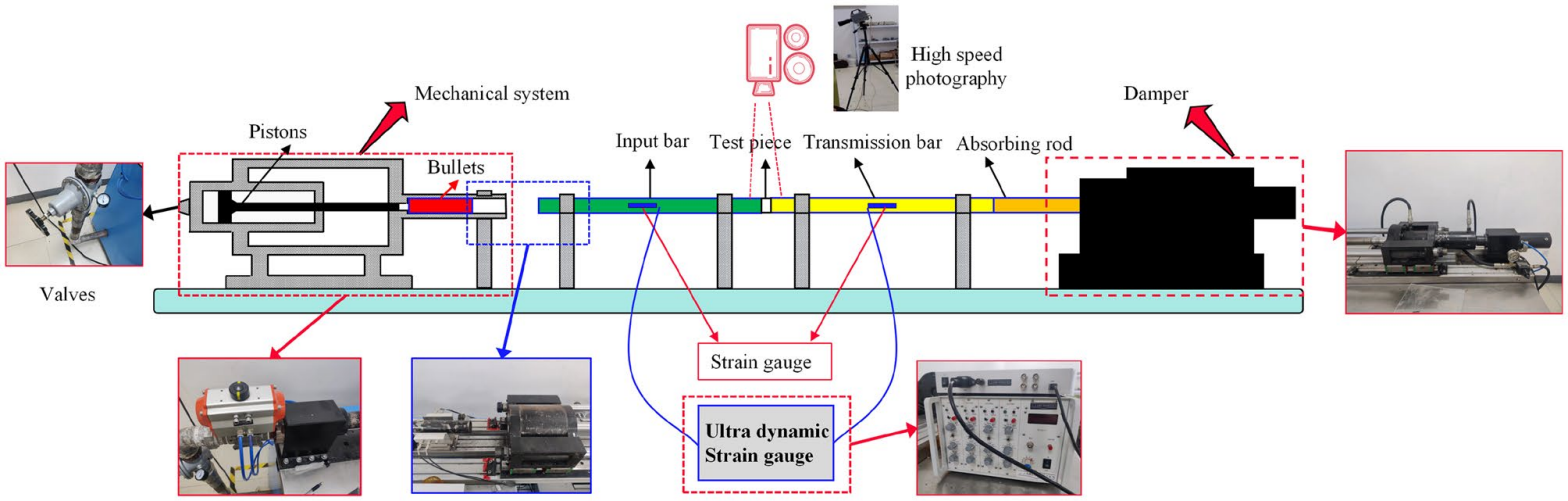
Split Hopkinson pressure bar experimental system.
  The parameters of the Split Hopkinson Pressure Bar apparatus are shown in Table 5.

1. SHPB experiment
  The standard specimens of sandstone are illustrated in Fig. 7. The Split Hopkinson Pressure Bar (SHPB) experimental apparatus was utilized, with its schematic diagram presented in Fig. 8. An impact air pressure of 0.15 MPa was chosen. During the SHPB experiments, a FASTCAM high-speed camera was used to record the crack propagation and final failure modes of the rock under dynamic impact. The camera's frame rate was set to 25,000 fps to observe the failure modes of the sandstone under impact.

**Table 5:** Main physical parameters of the projectile, incident bar, and transmission bar.

Parameter	Parameter value	Parameter	Parameter value
Diameter (mm)	50	P-wave velocity (m/s)	5410
Projectile length (mm)	400	Elastic modulus (GPa)	210
Incident bar length (mm)	2000	Poisson's ratio	0.28
Transmission bar length (mm)	2000	Density (kg/m^3^)	7810
2. Experimental results
  The stress–strain curves of sandstone under impact loading are depicted in Fig. 9. Three experimental trials yielded rock strengths of 97.73 MPa, 94.89 MPa, and 93.6 MPa, respectively, with an average strength of 95.41 MPa. The failure mode of the sandstone is shown in Fig. 10, where it has ultimately been completely fractured.

**Figure 9:**
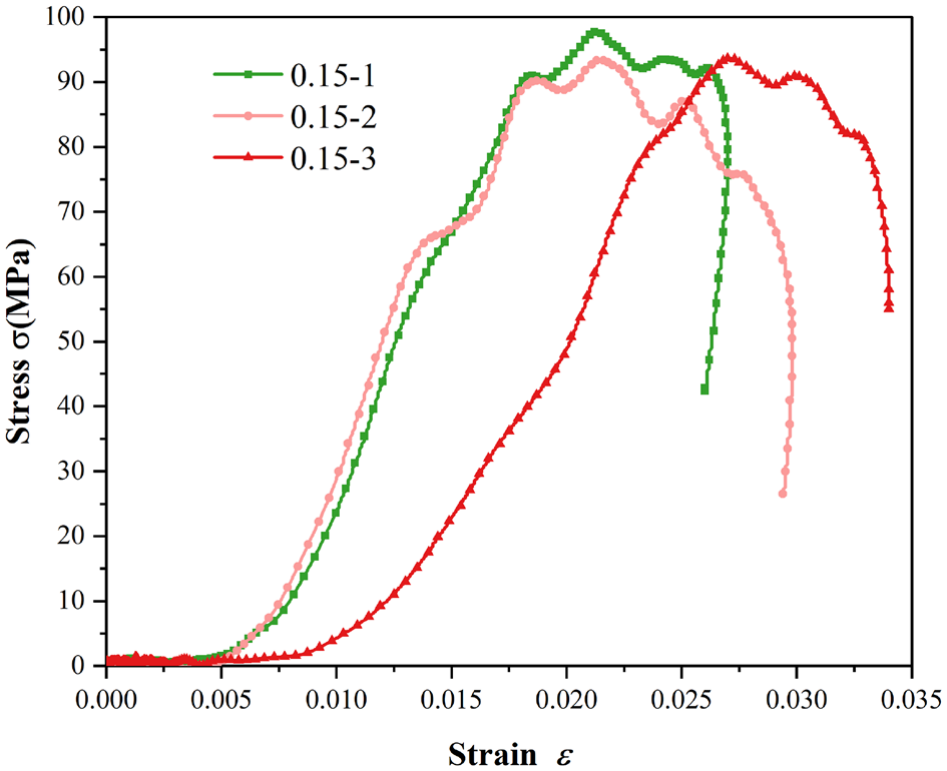
Stress–strain curve of rock under impact.

**Figure 10:**
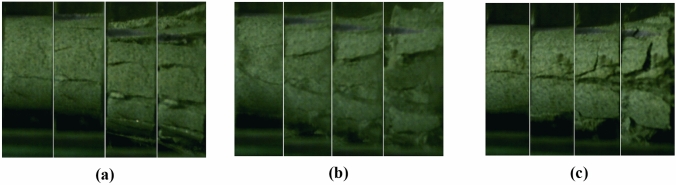
Damage effect at an air pressure of 0.15 MPa. (**a**) 0.15–1, (**b**) 0.15–2, (**c**) 0.15–3.
3. Numerical calculation model
  In this finite element simulation, the projectile length was set to 400 mm, with both the input and output rods measuring 2000 mm in length, and the specimen length was set to 25 mm. The diameter of both the rods and the specimen is 50 mm. During the contact process between the pressure bar and the specimen, an erosion algorithm was employed to analyze the face contact, and the friction effects that might occur at the contact interface were ignored. The projectile, incident rod, and transmission rod were all modeled using the same linear elastic material model, with the main physical properties outlined in the appendix. The constructed numerical model is presented in Fig. 11.

**Figure 11:**
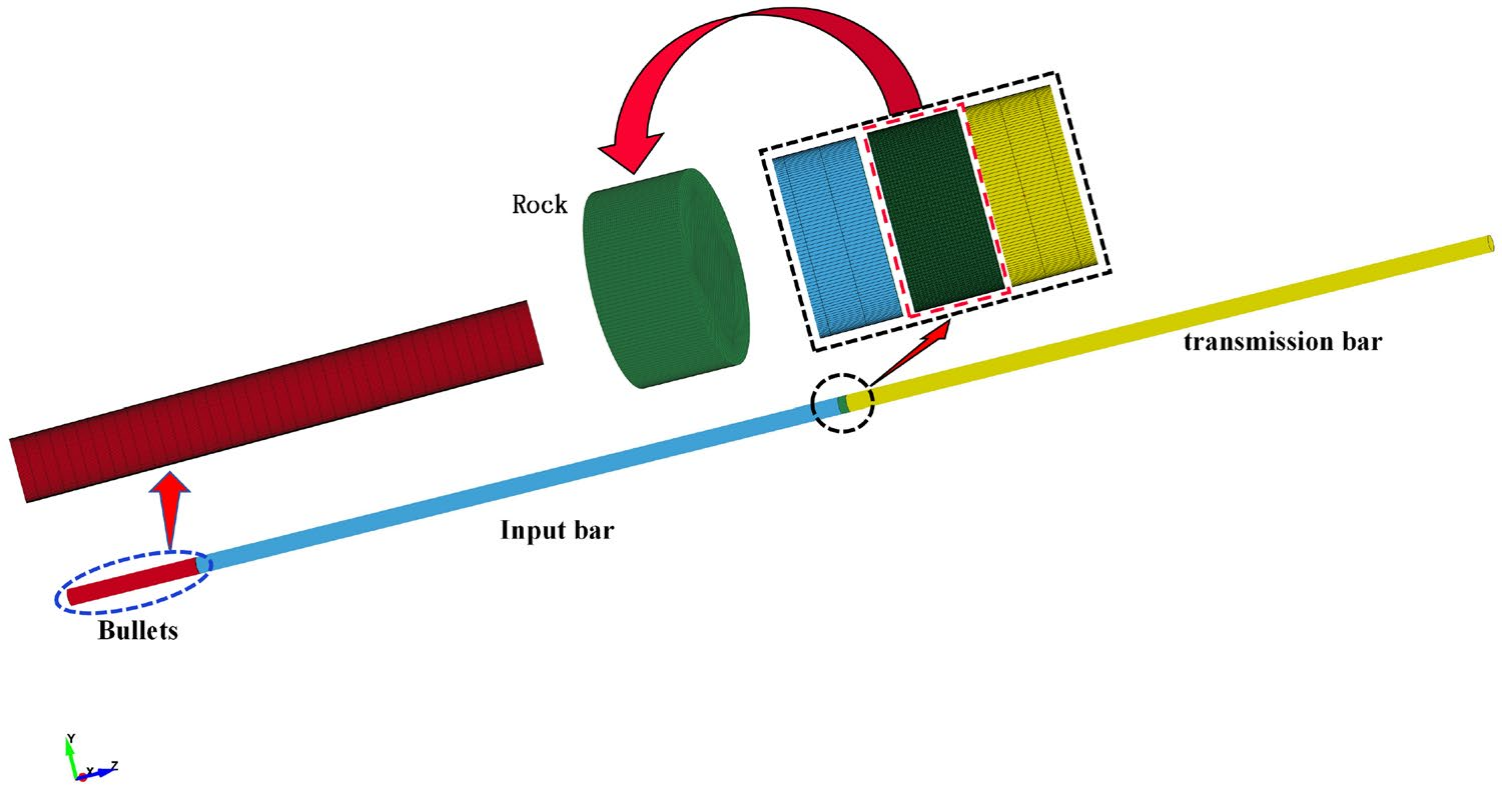
SHPB numerical model.
4. Numerical calculation results and comparison
  In this study, the model developed was applied to computational analysis in LS-DYNA, with the aim of verifying the applicability of the sandstone RHT constitutive model parameters. To ensure the accuracy of model validation, special attention was paid during the numerical simulation process to maintain the same shock air pressure conditions as in the experiments, ensuring that the numerical values of the shock air pressure were consistent with experimental data. As shown in Fig. 12, the calculation results demonstrate that the model's prediction of crack propagation phenomena largely agrees with experimental observations. Additionally, the strength performance of the rock also aligns with experimental outcomes, further corroborating the applicability and effectiveness of the sandstone RHT constitutive model parameters adopted in this study.

**Figure 12:**
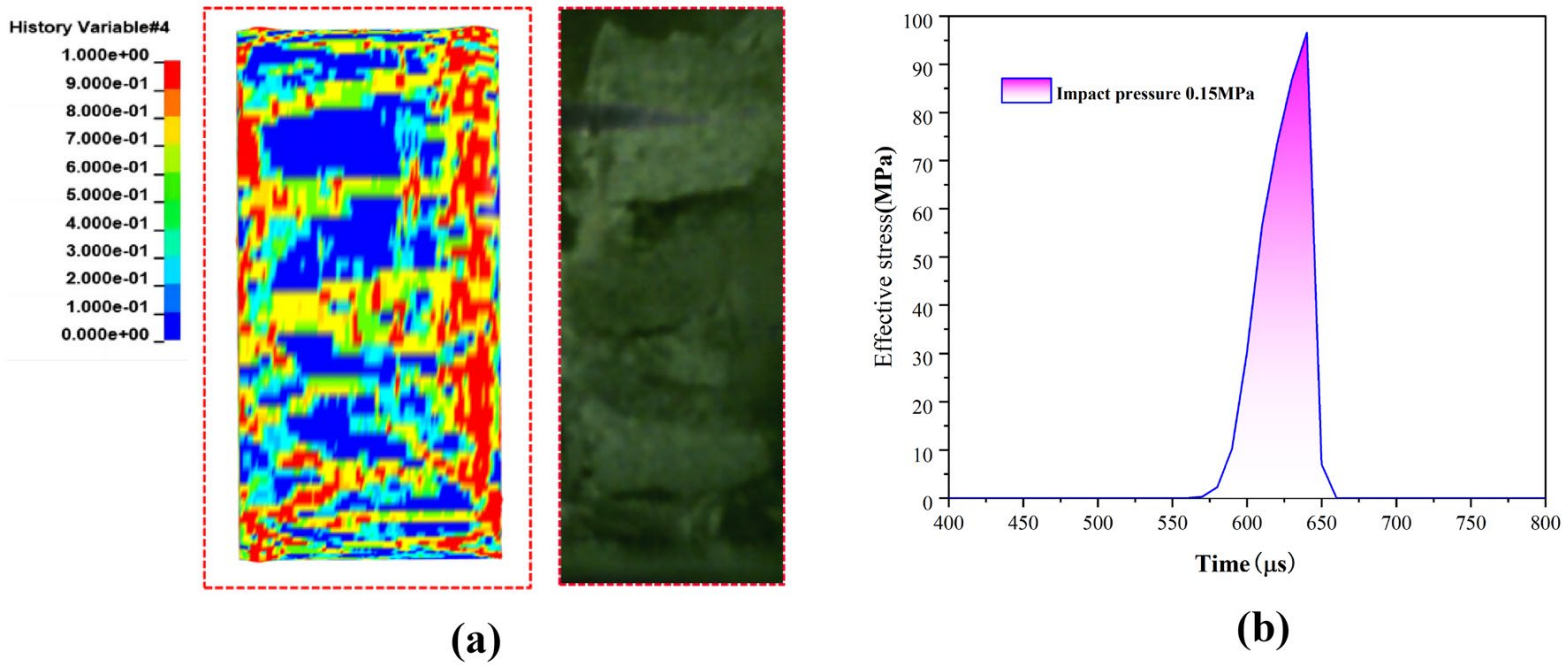
Comparison between computational results and experimental data. (**a**) Damage comparison diagram. (**b**) Stress comparison.

#### Other material parameters

Other material parameters can be found in Tables 6, 7, and 8^27^.

**Table 6:** Explosive parameters.

Density *ρ*_e_/kg·m^-3^	Detonation velocity *c*/m·s^-1^	*E*_e0_/GPa	Material parameters				
			*A*/GPa	*B*/GPa	*R*_1_	*R*_2_	*ω*
931	4160	3.87	494.6	18.91	3.907	1.118	0.333

**Table 7:** Air material parameters.

Density *ρ*_a_/kg·m^3^	Internal energy *E*_aQ_/Gpa	Initial relative volume *V*_a0_	Coefficients of the equation of state						
			*C*_0_	*C*_*1*_	*C*_*2*_	*C*_3_	*C*_4_	*C*_5_	*C*_6_
1.29	2.5e-6	1	0				0.4		0

**Table 8:** The stemmed material parameters in drill holes^37^.

Density/g·m^3^	Elastic modulus/Gpa	Poisson ratio
1.85	1.6e−4	0.3

### Results analysis

#### Surrounding rock damage analysis

**Figure 13:**
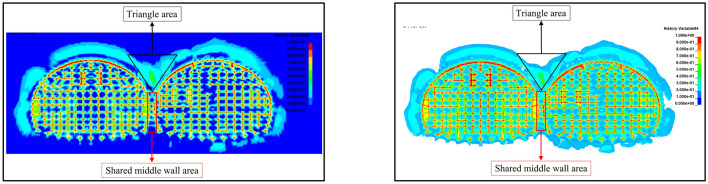
The blast damage effect.

The numerical simulation results are shown in Fig. 13, which shows that when the original blasting scheme is used in the continuous arch tunnel without intermediate pilot tunnels, the impact of tunnel blasting and excavation on the tunnel surrounding rock is significant. The damage range of the surrounding rock at the arch top, right arch shoulder, arch foot, and arch bottom of the first tunnel was extensive with a maximum damage depth of 2.48 m, and the influence of surrounding rock at the left arch shoulder was the smallest; the damage range of surrounding rock at the arch top, arch shoulder, and arch foot of the following tunnel was also extensive with a maximum damage depth of 2.94 m. The influence of the arch waist on the right side was the smallest. In particular, regarding the region of the shared center wall and the cross region of the arch of the continuous arch tunnel, the damage generated in the region by blasting and excavation of the first tunnel and blasting and excavation of the following tunnel formed a superimposed cumulative effect, thereby resulting in excessive damage values in the region.

The maximum damage variable D in the region of the shared center wall was close to 1, and the maximum damage variable D in the region of the arch intersection of the double-arch tunnel was 0.41. As shown in the previous section, it was known that the damage threshold of the rock body was *D*_*1*_ = 0.19. Therefore, after the successive blasting and excavation of the first and the following tunnel of the cave, the tunnel's retaining rock body had already been damaged. A great deal of fissures were produced in the surrounding rock of the triangular region above the shared center wall, which may be a result of the tunnel-reserved rock body being unstable. The peripheral rock at the location of the shared center wall may, in the future, collapse; thus, it is necessary to analyze the damage pattern in this area.

**Figure 14:**
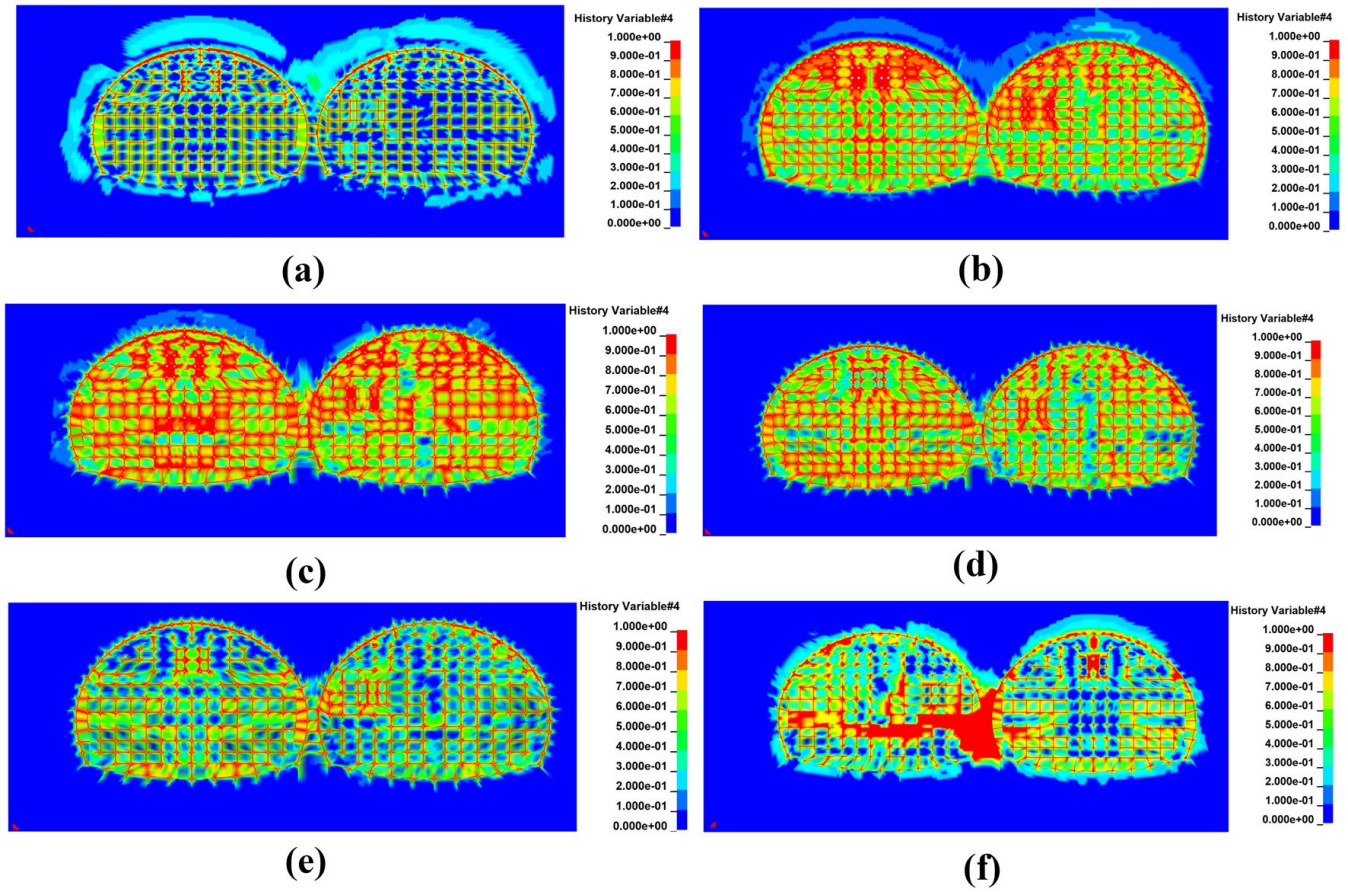
The damage map of the tunnel cross-section at different blasthole depth locations. (**a**) At a 0 cm blasthole depth. (**b**) At a 30 cm blasthole depth. (**c**) At a 60 cm blasthole depth. (**d**) At a 90 cm blasthole depth. (**e**) At a 120 cm blasthole depth. (**f**) At a 150 cm blasthole depth.

After blasting and excavating the first and following tunnel successfully, the damage range of the rock body at different cross-section locations along the tunnel excavation direction was found to be diverse. From Fig. 14, it can be seen that, from the tunnel excavation palm face to the excavation direction, the damage range of the rock body was from large to small and then from small to large. The damage range of the rock mass on the cross-section at the tunnel palm face was found to be the largest. Due to the existence of the critical surface, the wave impedance of the air was much smaller than the wave impedance of the rock body, thereby resulting in the propagation of the critical surface of almost all of the stress waves to the form of tensile stress wave reflection^38^. This enhanced the effect of the blasting and thus increased the scope of the damage caused by the blasting.

From the previous description and analysis, it can be seen that the damage range located at the tunnel palm face was the largest, and the damage generated in the upper triangle of the shared center wall was the smallest. For a continuous arch tunnel without a middle pilot tunnel, the weakest location was the connection between the preliminary contact point of the first tunnel's first support and the following tunnel's initial support. Therefore, it is necessary to analyze the damage evolution law of the rock body above the shared center wall in the blasting and excavation process of the first and following tunnel.

**Figure 15:**
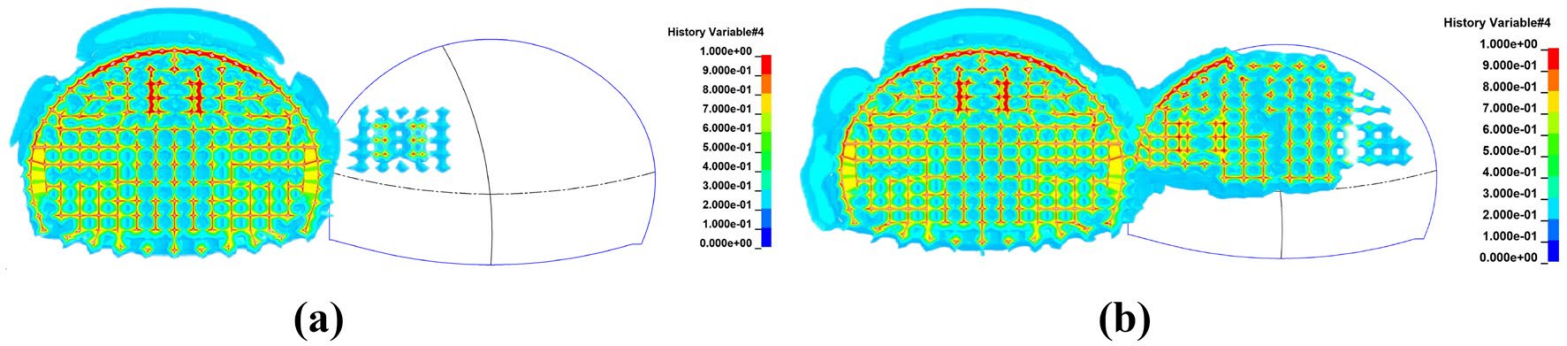
Depicts the damage to the surrounding rock in the cross-sectional area of the arch parts of the double-arch tunnel at different stages. (**a**) During the blasting of the left guide tunnel on the upper step of the following tunnel. (**b**) During the blasting of the right guide tunnel on the upper step of the following tunnel.

As illustrated in Fig. 15a, the initiation of blasting has begun in the left pilot tunnel on the upper bench behind the first tunnel. It is observed that the prior excavation by blasting in the leading tunnel has caused damage to the surrounding rock in the cross-section area of the intersecting arch sections of the double-arch tunnel. Additionally, some degree of damage has also occurred to the rock surrounding the pilot holes in the vicinity of both the left pilot tunnel on the upper bench and the holes around the lower bench behind the advancing tunnel. Figure 15b shows that the blasting in the right pilot tunnel on the upper bench behind the advancing tunnel has started. Following the blasting in the left pilot tunnel, the damage to the surrounding rock in the cross-section area of the intersecting arch sections of the double-arch tunnel has intensified, leading to accumulated damage on the pre-existing conditions. Notably, the rock near the lower bench, adjacent to the side of the leading tunnel, has also experienced certain damages, with the rock damage near the median diaphragm area effectively becoming continuous.

**Figure 16:**
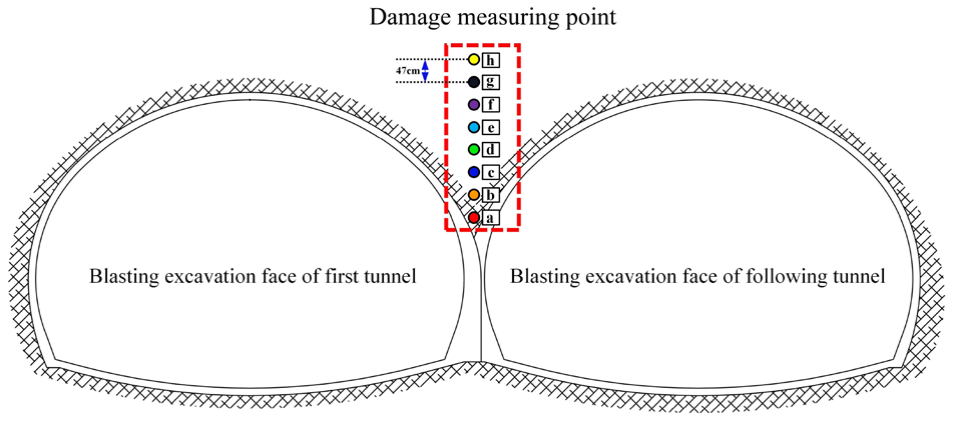
The detection points of the surrounding rock damage in the arch cross-region of the continuous arch tunnel.

To clearly and intuitively observe the changes in the surrounding rock damage within the cross-sectional area of the arch parts of the double-arch tunnel, the approach begins from a position close to the shared central wall of the tunnels. Eight surrounding rock damage monitoring points are selected upwards at equal intervals. The data reflecting changes in damage at these monitoring points are extracted. The schematic diagram of these monitoring points is illustrated in Fig. 16.

**Figure 17:**
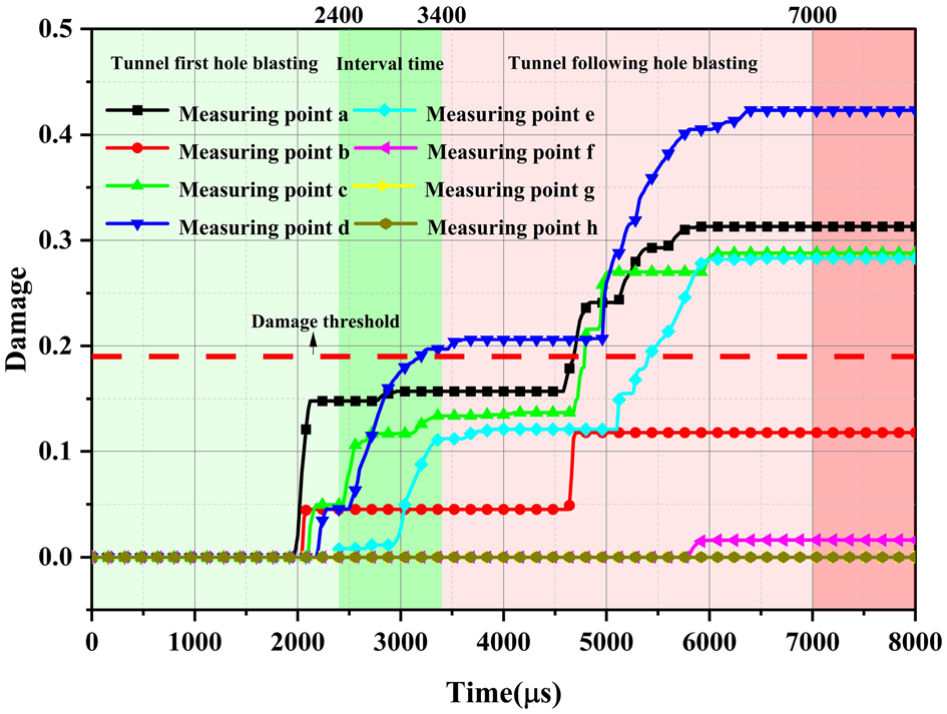
Measurement point damage.

The results of this are shown in Fig. 17. Furthermore, in the process of the blasting and excavation of the first and following tunnel successfully, there was a certain pattern in the damage produced by the surrounding rock in the cross-region of the arch of the continuous arch tunnel: with the increase in the distance from the measuring point to the shared center wall, the damage of the measuring point first shows a decrease, then increase, and—finally—a decrease again (until it becomes zero). The damage to the surrounding rock in the cross-region of the arch of the continuous arch tunnel was caused by the blast excavation of the first tunnel, which was found to be slight and did not reach the damage threshold of the tunnel rock. From the figure, it can also be seen that the explosions, via explosives, in the MS17 and MS25 sections (the following tunnel) had the greatest impact on the damage to the surrounding rock in the arch cross-region of the continuous arch tunnel. The main reason for this was that the blast center distance was small, and the number of starting explosives was large. When the following tunnel blasting excavation was being conducted, the damage at the perimeter rock in the arch cross-region of the continuous arch tunnel produced a cumulative effect. The MS1-31 section explosives produced a large degree of damage to the surrounding rock in the arch cross-region of the continuous arch tunnel when compared with the damage produced by the first tunnel blasting, i.e., an increase of 193%. The main reason for this was, still, the closer distance and the larger number of starting explosives.

#### Effective stress analysis of initial support

When performing tunnel blasting excavation, the blasting action will exert effective stress on the support structure. If the effective stress is too large, it will have adverse effects on the support structure; in addition, it may cause the support structure to crack or the shotcrete to fall off. Therefore, it is necessary to analyze the effective stress of the initial support structure.

**Figure 18:**
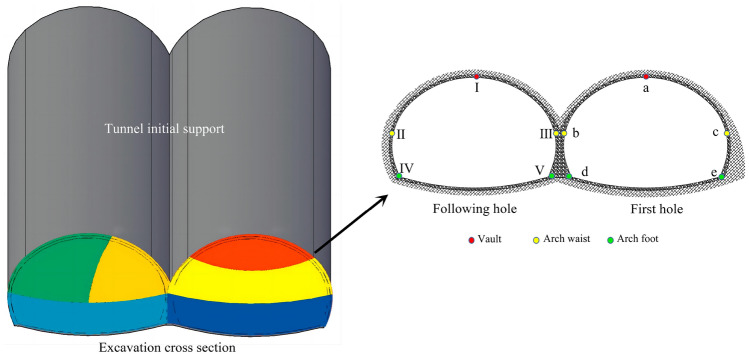
Schematic of the effective stress monitoring location of the initial support.

**Figure 19:**
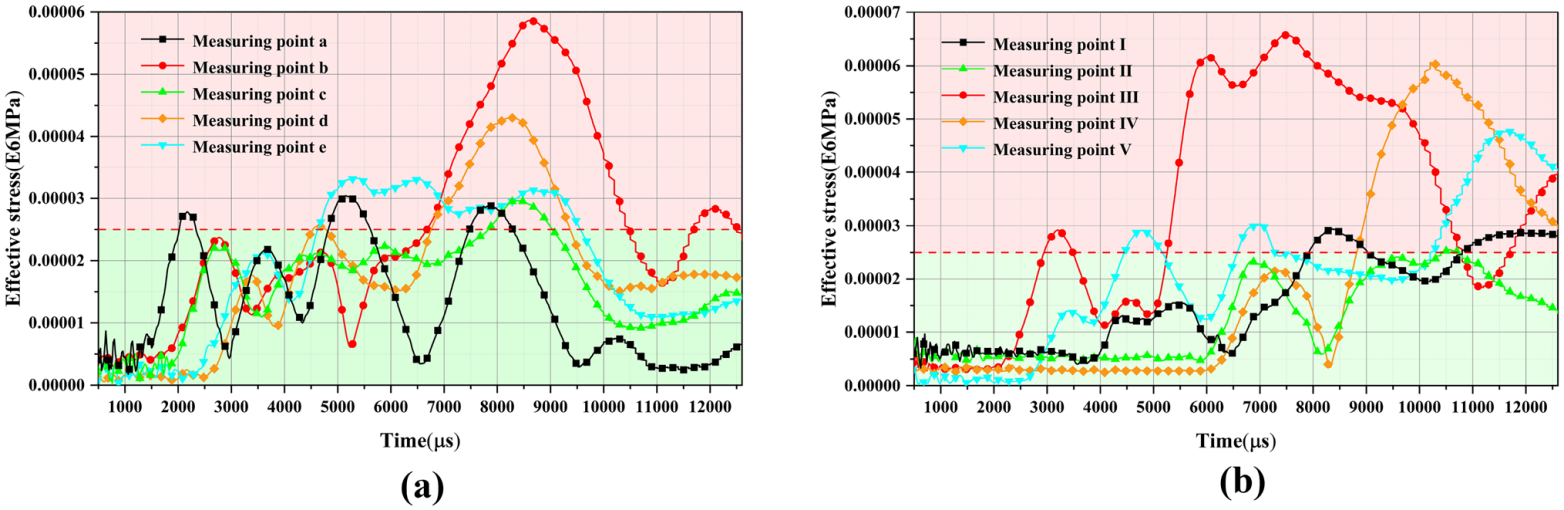
Calculation results of the effective stress at the measurement points of an initial support structure. (**a**) Effective stress at the first tunnel measurement point. (**b**) Effective stress at the following tunnel measurement point.

The effective stresses of the initial support structure adjacent to the palm face were analyzed. The location of the monitoring point arrangement is shown in Fig. 18. The monitoring points were at the top of the arch, the arch waist, and at the foot of the arch. Moreover, the location of the arch waist was set according to the intersection of the upper and lower steps of the CD method. The numerical simulation results of the effective stress of the initial support structure are shown in Fig. 19. When the original blasting scheme was used for the blasting excavation of a multi-arch tunnel without a middle pilot tunnel, the arch foot, vault, and arch waist of the supporting structure of the first hole and the following hole all exceeded the strength that the initial support concrete could bear, especially with respect to the position of the arch waist and arch foot near the middle partition wall. More specifically, the maximum effective stress was more than 160% of the strength that the initial support concrete could bear. The reasons for the initial support structure to produce large effective stress were analyzed, and the following reasons were considered to have caused the effective stress of the initial support structure to be large: the original blasting program of the single section of the detonation of explosives was too large, especially with respect to the peripheral holes of the charge, which was the closest to the initial support structure. It can also be seen from the results that the blasting and excavation of the continuous arch tunnel without a middle pilot tunnel had a greater impact on the shared center wall.

## Optimization of blasting solutions

**Figure 20:**
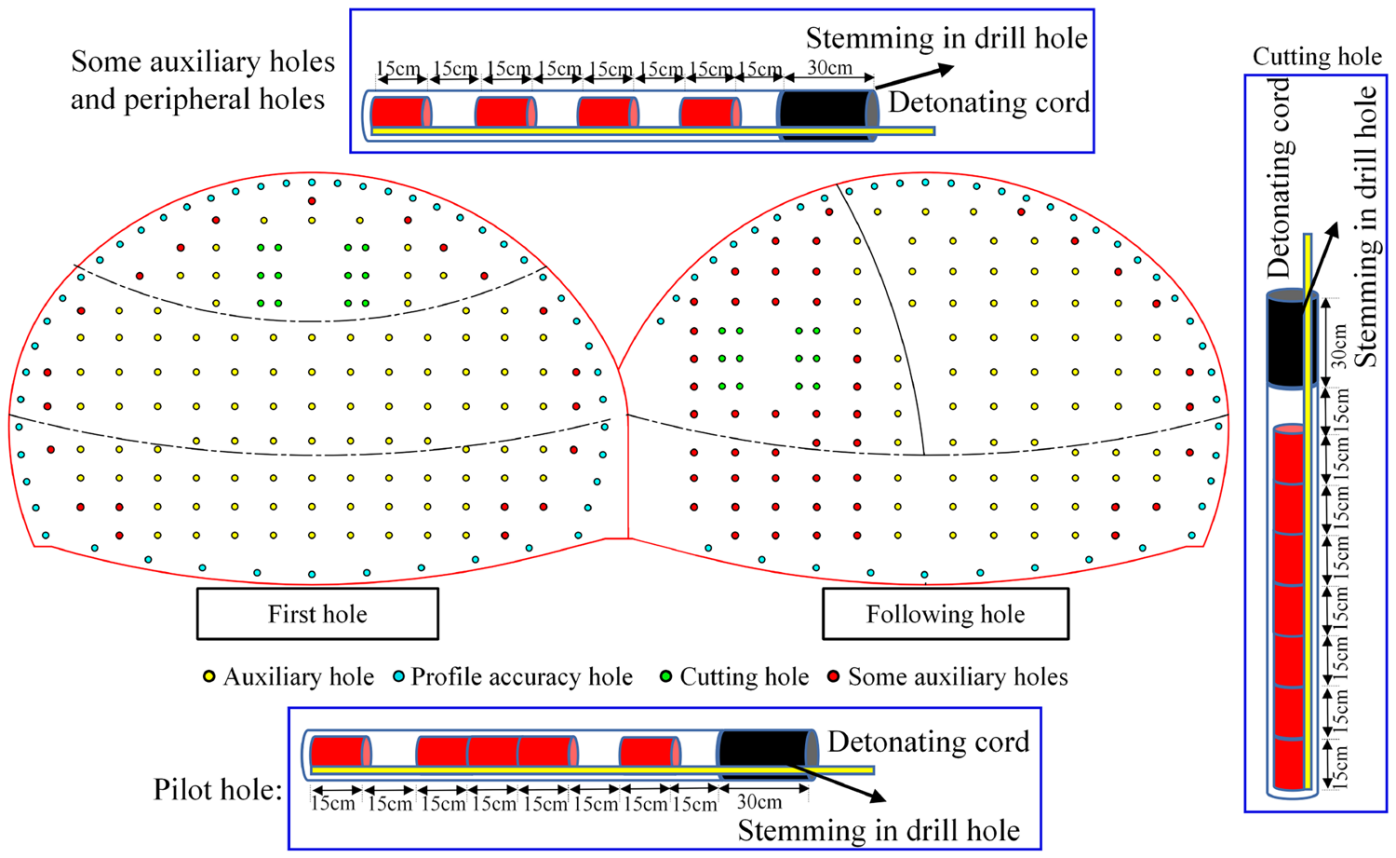
The optimized blasting plan charging schematic diagram.

**Figure 21:**
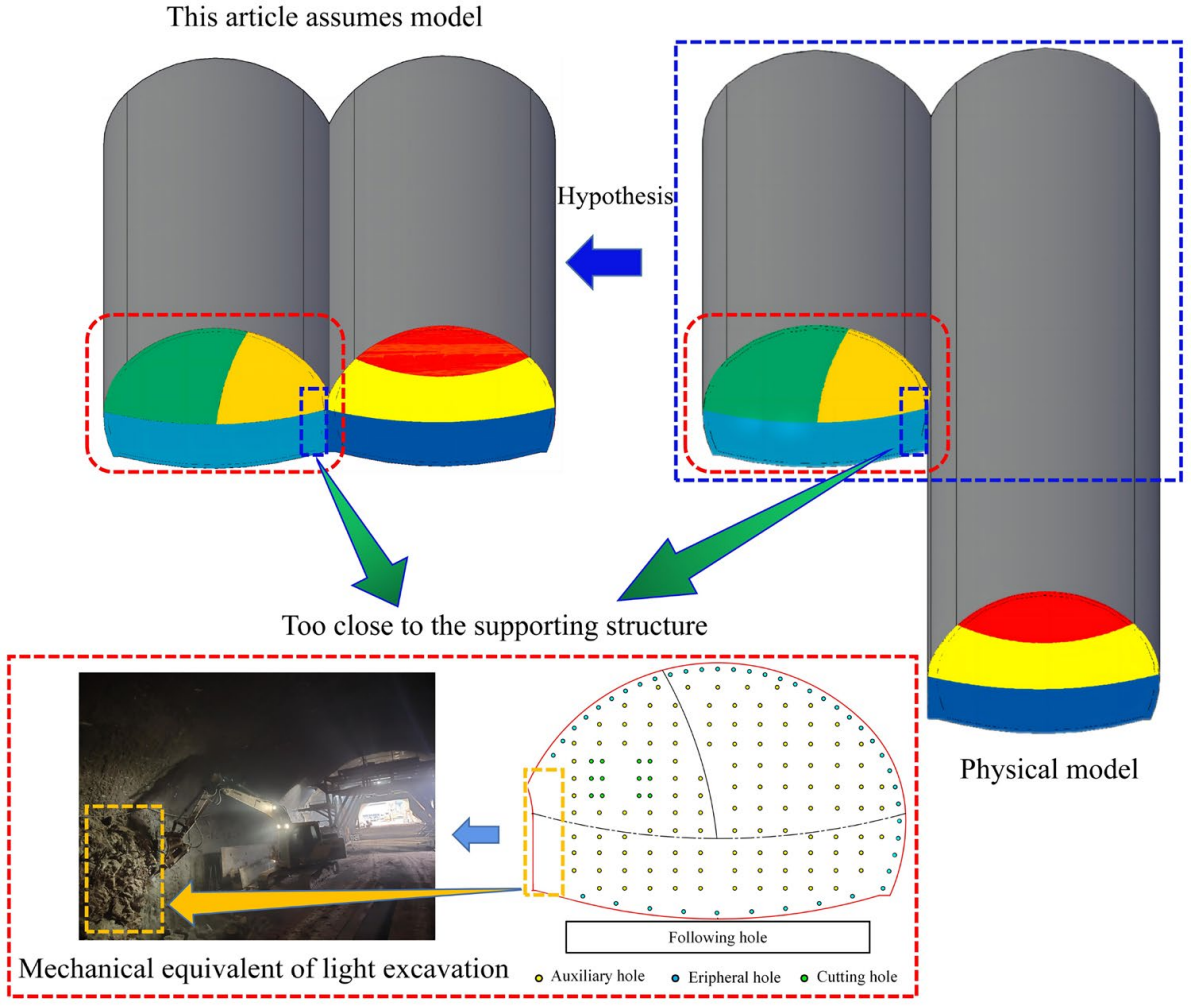
Optimization of the gun holes on the side of the following tunnel that was adjacent to the first tunnel.

The original blasting program was optimized according to the analysis that was conducted on the numerical simulation results. Considering that the charge of the blasthole was the main factor affecting the tunnel's blasting effect and the support structure's mechanical response^39^, this study only focused on the charge of the blasthole with respect to optimizing the blasting scheme. The length of one section of the emulsion explosives was 30 cm. To optimize the loading of the holes more effectively and accurately, one section of the emulsion explosives was divided into two sections. One of these sections was 15 cm, as shown in Fig. 20: (a) The adjustment of the loading of the holes was carried out according to the analysis of the results of the numerical calculations, and the stemming in the drill hole was increased by 15 cm to 30 cm. The loading of the peripheral holes in the single holes was reduced to 0.6 kg, which reduced the explosives volume by 56%. The charge of the single hole of the peripheral hole was reduced to 0.6 kg, which reduced 56% of the explosives and changed to interval charging. In addition, the charge of the single hole of the auxiliary hole was reduced to 0.750 kg, which reduced 44% of the explosives and changed to interval charging. Moreover, since part of the auxiliary holes were closer to the supporting structure, the charge of them was reduced to the same as that of peripheral holes. (b) The gun holes on the side of the following tunnel near the first tunnel were optimized (as shown in Fig. 21). This was conducted because, in the actual project, the distance from the palm surface of the following tunnel blasting excavation to the supporting structure of the first tunnel was especially close, and if it were not optimized for the gun holes in the following tunnel, the blasting excavation of the following tunnel would have damaged the supporting structure of the first tunnel, thus leading to cracking of the supporting structure of the concrete and to the occurrence of a safety accident. As such, the two rows of gun holes in the following tunnel near the first tunnel were omitted (they were about 1.9 m)^1^. (c) The charge of some of the auxiliary holes in the upper left step of the following tunnel of holes was changed to the same charge as that of the peripheral holes, i.e., a single-hole charge of 0.6 kg; thus, there was a reduction in the charge by 56%.

### Surrounding rock damage

**Figure 22:**
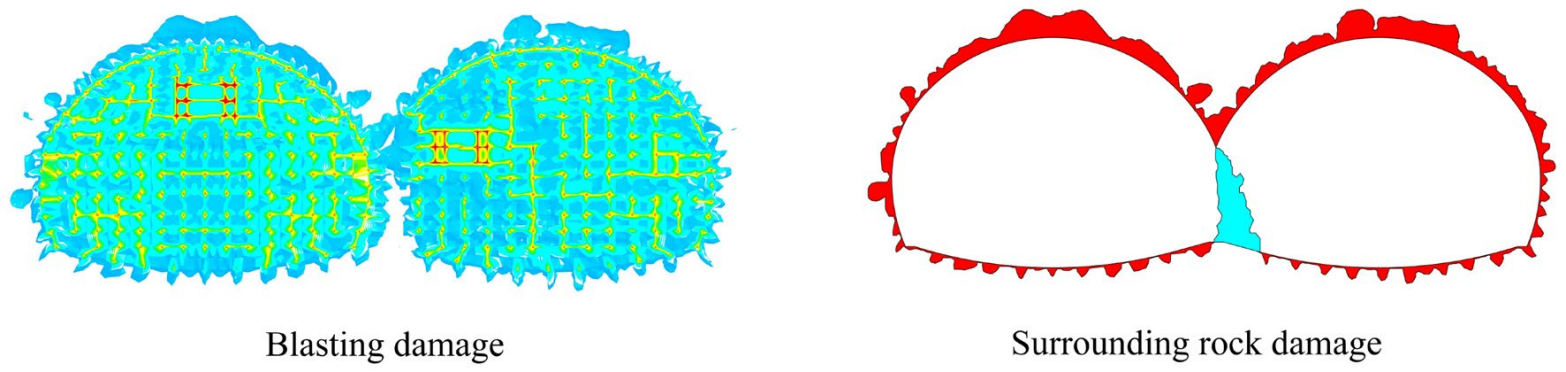
Surrounding the rock damage map after optimization of the blasting scheme.

The findings from the numerical simulation that were obtained upon optimizing the blasting scheme can be observed in Fig. 22, and they indicate a considerable reduction in the damage to the surrounding rock. Specifically, the top of the arch experiences the highest degree of damage, but this was reduced by 53% when compared to the pre-optimized scheme. Additionally, in the cross-region of the double-connected arch tunnel arch, the damage range of the surrounding rock was reduced by 67% when compared to the scheme before optimization. By subtracting some of the blastholes in the following tunnel of holes near the side of the shared center wall, no damage occurred to the rock in this area. Furthermore, in order to minimize the impact of the blasting excavation on the support structure, mechanical excavation was conducted in this area (which was about 1.9 m wide).

### Effective stress in the initial support

**Figure 23:**
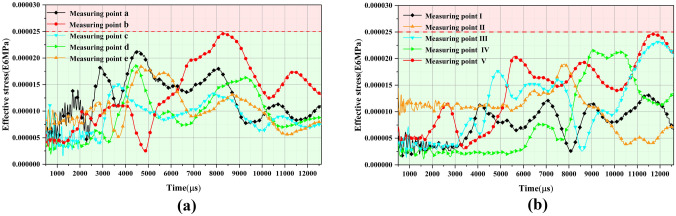
Calculation results of the effective stress at the measurement points after optimization of the scheme. (**a**) Effective stress at the first tunnel measurement point. (**b**) Effective stress at the following tunnel measurement point.

The measurement points of the effective stress of the supporting structure after optimizing the blasting scheme were consistent with those before the optimization. The numerical calculation results are shown in Fig. 23. Furthermore, the optimized blasting scheme had a significant reduction in the effective stress of the supporting structure, and these all met the design requirements. Compared with the original blasting scheme, the peak effective stress of the supporting structure of the first tunnel was reduced from 29.7 to 57.9%, and the effective stress of the supporting structure of the following tunnel was reduced from 25.9 to 64.8%.

## Example of the Xiqu district tunnel project

### Blasting effect

**Figure 24:**
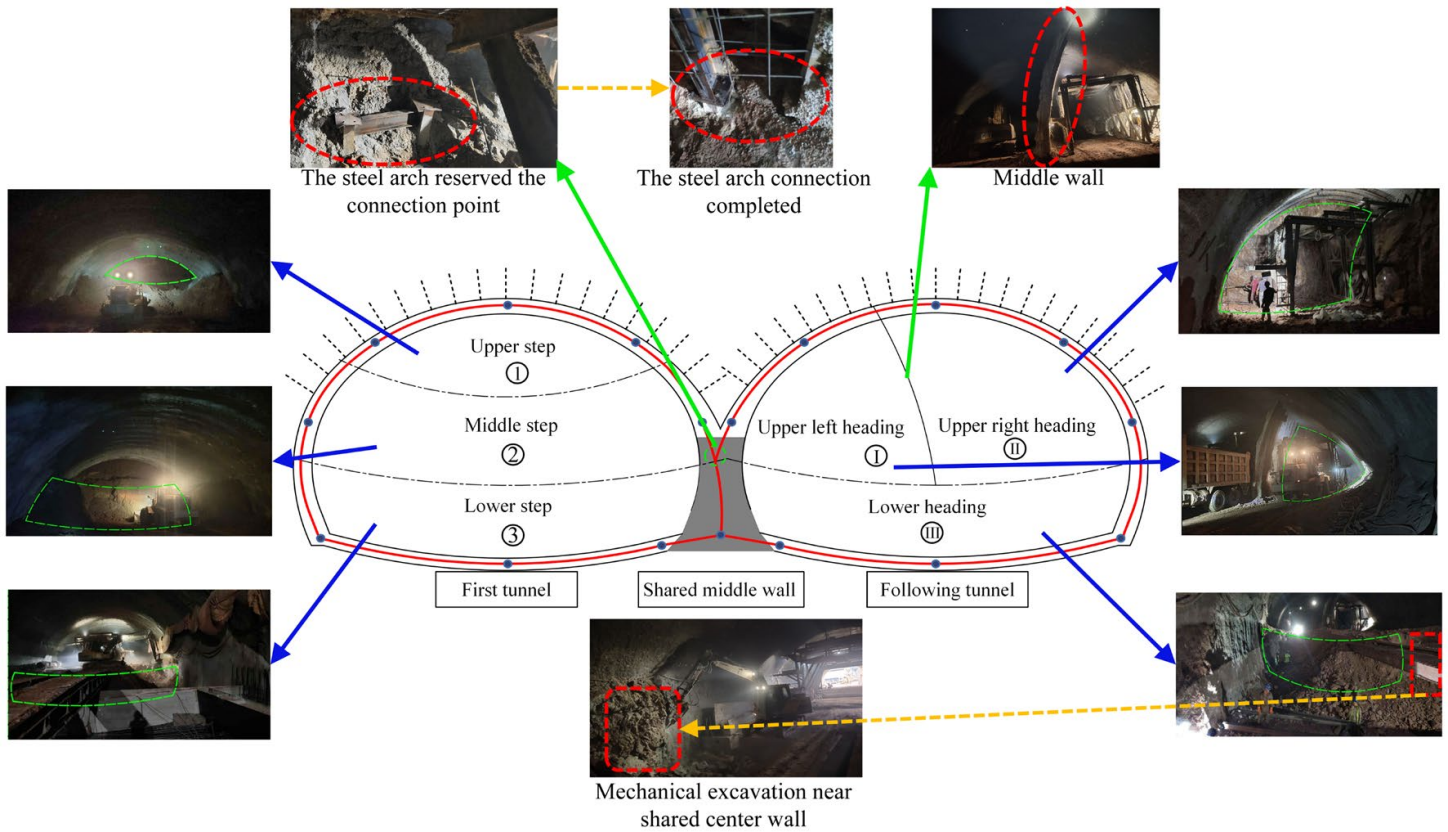
Schematic diagram of the on-site construction of a continuous arch tunnel without a middle pilot tunnel.

The on-site engineering adopted an optimized blasting scheme for the double-arch tunnel, with each advancement distance being 1.5 m. The excavation areas for the upper, middle, and lower benches of the leading tunnel were 39.41 m^2^, 68.81 m^2^, and 63.35 m^2^, respectively. The total explosive quantities for excavating each bench were 35.25 kg, 48.30 kg, and 44.25 kg, respectively. The explosive consumption per cubic meter of rock for each bench was 0.89 kg, 0.70 kg, and 0.70 kg, respectively. For the trailing tunnel, the excavation areas of the right upper guide tunnel, left upper guide tunnel, and lower guide tunnel were 45.49 m^2^, 61.87 m^2^, and 54.80 m^2^, respectively. The total explosive quantities for excavating each of these areas were 34.95 kg, 44.25 kg, and 36.15 kg, respectively. The explosive consumption per cubic meter of rock for each of these benches was 0.77 kg, 0.72 kg, and 0.66 kg, respectively. Moreover, following the blasting excavation under working condition four, a portion of the tunnel face rock near the arch waist to the arch foot of the trailing tunnel, approximately 2.0 m wide, did not undergo blasting. Therefore, mechanical excavation was employed on-site, as shown in Fig. 24.

### Blasting overbreak analysis for continuous arch tunnels

**Figure 25:**
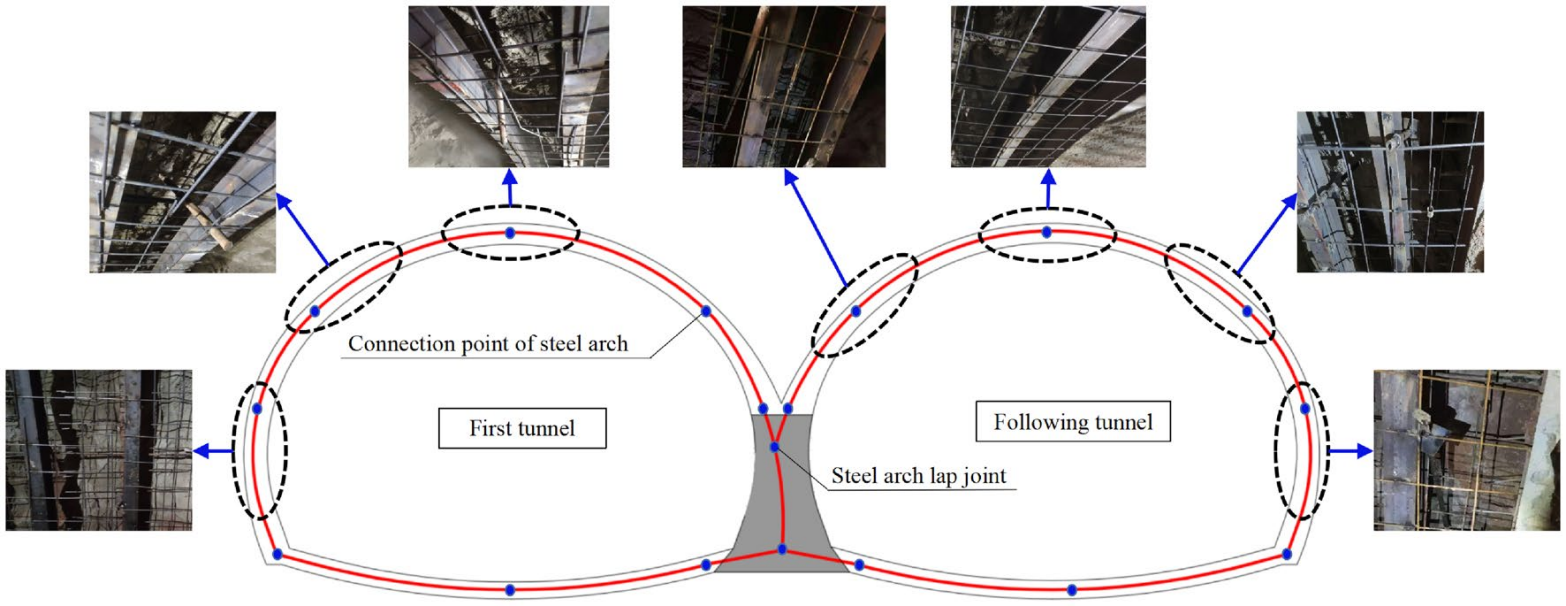
Erection of steel arches in continuous arch tunnels and over-excavation of surrounding rock.

After the blasting in the experimental section was conducted following the optimized blasting plan, the effects of overbreak in local surrounding rock and the installation of steel arch frames in the continuous arch tunnel are illustrated in Fig. 25. It is observable that the overbreak of the surrounding rock at the crown and shoulder sections of the arch tunnel is significant. To visually observe the overbreak and underbreak conditions, the overbreak and underbreak of the tunnel were measured and statistically analyzed.

**Figure 26:**
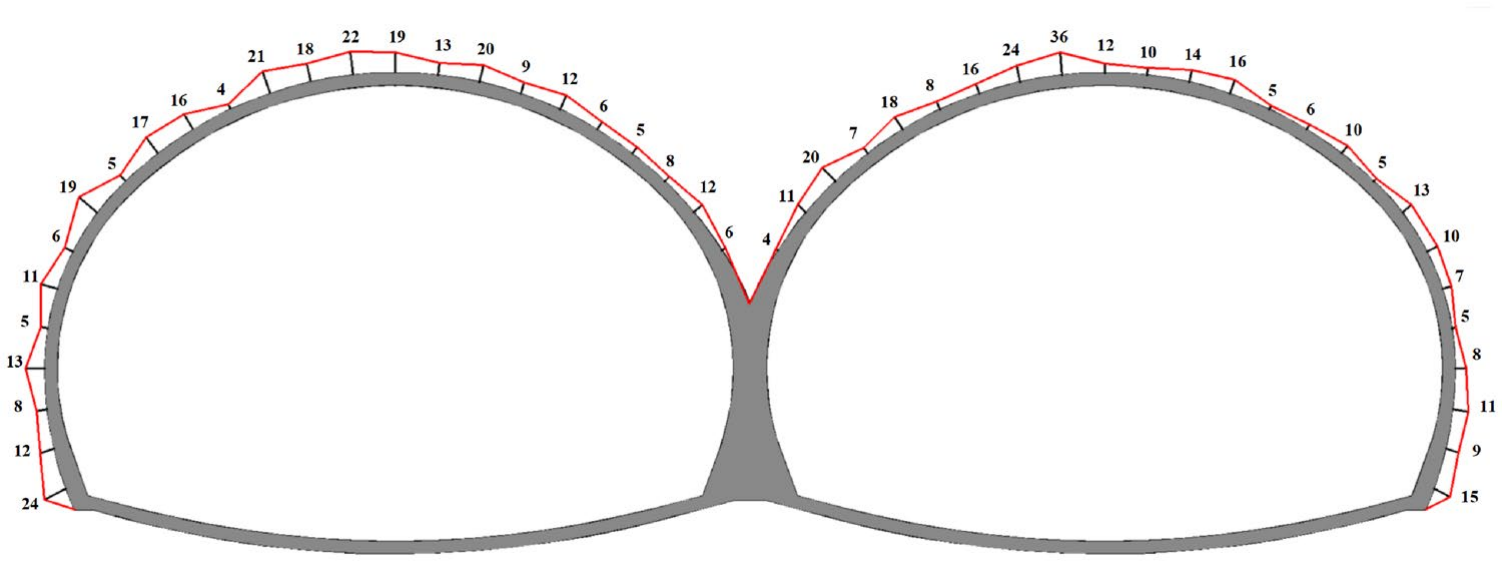
Statistical charts of over-excavation in continuous arch tunnels.

**Figure 27:**
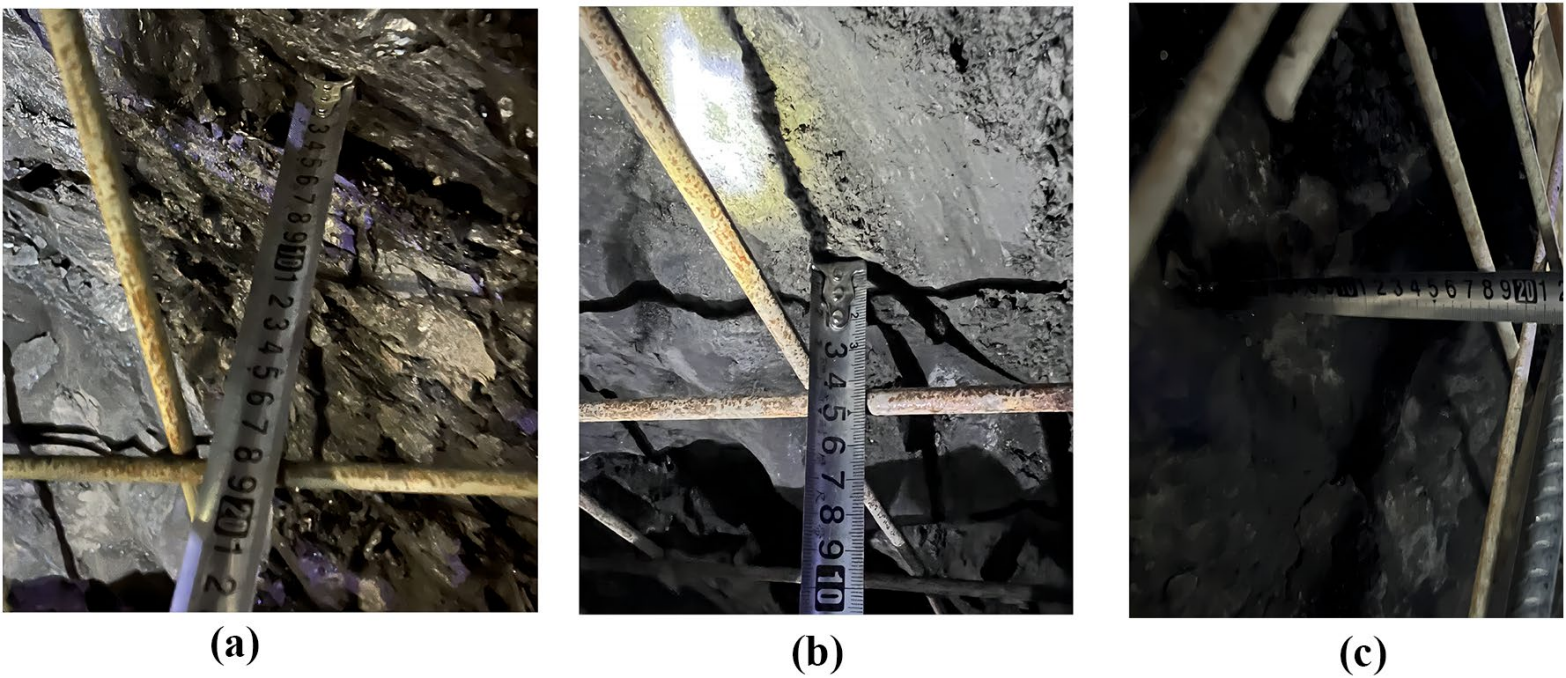
Local over-excavation measurements at different positions in continuous arch tunnels. (**a**) Arch waist of the following tunnel. (**b**) Arch shoulder of the leading tunnel. (**c**) Arch waist of the leading tunnel.

The statistical results of the tunnel overbreak are shown in Figs. 26 and 27. The results indicate that the advance tunnel exhibits significant overbreak at the crown, arch shoulders, and the left arch waist, while the follow-up tunnel also shows considerable overbreak at the arch shoulders and crown. Despite instances of excessive local overbreak exceeding the values permitted by the standards, overall, neither the advance tunnel nor the follow-up tunnel experienced any underbreak, with an average overbreak of 11.50 cm, meeting the design specifications. The reason for the excessive local overbreak is that workers drilling peripheral holes around the tunnel face cannot always maintain a strict 90-degree angle perpendicular to the face. This results in the peripheral holes having an outward inclination, causing the explosive charges to shift towards one side of the surrounding rock. To prevent excessive overbreak of tunnel surrounding rock due to the outward inclination of peripheral holes, it is possible to adjust the position of the peripheral holes slightly towards the center of the tunnel face.

## Conclusion

In an attempt to address the shortcomings of the traditional double-arch tunnel construction method, such as overly complex processes and a long construction period, this study proposes the construction method of a continuous arch tunnel without a middle pilot tunnel. This new method omits the construction of a middle pilot tunnel and shortens the construction distance between the left and right tunnels, which leads to a more complex impact in terms of blasting and excavating continuous arch tunnels with respect to the surrounding rock and supporting structure. In addition, it is of great significance in terms of investigating the impact law on the engineering design and construction. This study utilized LS-DYNA finite element software to examine the peripheral rock damage law and the dynamic response characteristics of the supporting structure after blasting in a double-arch tunnel. Based on the results of this study, the blasting program was optimized to improve the reliability of the construction method of the continuous arch tunnel when constructed without a middle pilot tunnel, and this optimized blasting program was then applied to the project site.

Based on the results of the obtained research, we drew the following main conclusions:

1. Under the original blasting program, due to the excessive loading of the gun holes and the short distance between the left and right tunnels, the surrounding rock at the tunnel arch, arch shoulder, and foot of the arch suffered an extensive range of damage; moreover, the effective stress of the supporting structure exceeded the strength design value. It was particularly the arch cross-region of the continuous arch tunnel in which the damage range of the surrounding rock was the most serious, and it was also there that the phenomenon of damage accumulation occurred. Meanwhile, the effective stress generated at the shared center wall reached its maximum value, i.e., exceeding 160%.
2. The blasting scheme was optimized in accordance with the findings obtained from the analysis of the numerical simulation results. To reduce the mutual influence of the left and right hole blasting excavations, the blasthole of the following tunnel near the side of the first tunnel was omitted and mechanical excavation was used (with a width of about 2 m). In this way, the lateral distance between the left and right hole excavation disguisedly increased. After the optimization of the blasting program, the maximum damage range occurred at the top of the arch—specifically, a reduction of 53%. The surrounding rock damage range in the cross-region of the arch was reduced by 67%; in addition, there was a reduction from 25.9 to 64.8% with respect to the effective stress of the support structure in meeting the design requirements.
3. The model established in this research focused on a study of the surrounding rock damage law and the effective stress of the supporting structure in the same cross-section of the first and the following tunnel without a middle pilot tunnel. Conversely, in the actual project, the palm surface of the first and the following tunnel could not exist in the same cross-section. As such, different construction steps could not be conducted in the same cross-section. Therefore, we will conduct a subsequent study for those aspects that were or could not be considered in this research.

## Data Availability

The datasets used during the current study available from the corresponding author on reasonable request.
